# Combined metabolome and transcriptome analyses reveal that growing under Red shade affects secondary metabolite content in Huangjinya green tea

**DOI:** 10.3389/fgene.2024.1365243

**Published:** 2024-04-10

**Authors:** Zaifa Shu, Qingyong Ji, Tianjun He, Dayun Zhou, Shenghong Zheng, Huijuan Zhou, Weizhong He

**Affiliations:** Lishui Institute of Agricultural and Forestry Sciences, Lishui, Zhejiang, China

**Keywords:** shading nets, green tea, caffeine, flavonoids biosynthesis, phytohormone signalling, phenylpropanoid biosynthesis

## Abstract

Shading treatments impact the tea (*Camellia sinensis* L.) quality. The sunlight sensitive varieties can be grown under shading nets for better growth and secondary metabolite content. Here, we studied the responses of a sunlight sensitive green tea variety “Huangjinya” by growing under colored shading nets (red, yellow, blue, and black (75% and 95%) shading rates) to find out the most suitable color of the shading net. Red shading was the most promising treatment as it positively affected the weight and length of 100 one-bud-three leaves and reduced the degree and rate of new shoots burn compared to control (natural sunlight). We then explored the comparative metabolomic changes in response to red shading by using UPLC-ESI-MS/MS system. The amino acids and derivatives, flavonoids, and alkaloids were downaccumulated whereas lipids, organic acids, and lignans were upaccumulated in Red shade grown tea samples. The red shading nets caused a decreased catechin, epicatechin, dopamine, and L-tyramine contents but increased caffeine content. We then employed transcriptome sequencing to find key changes in expressions of related genes and pathways. Notably, key genes associated with the phenylpropanoid and flavonoid biosynthesis pathways exhibited complex regulation. These expression changes suggested a potential trend of polymerization or condensation of simple molecules like catechin or pelargonidin into larger molecules like glucoside or proanthocyanidins. Here, Red shading net triggered higher expression of genes enriched in lipid biosynthesis and jasmonic acid biosynthesis, suggesting an interplay of fatty acids and JA in improving tea performance. These findings contribute to the metabolic responses of Huangjinya tea to red shading nets which might have implications for flavor and health benefits. Our data provide a foundation for further exploration and optimization of cultivation practices for this unique tea variety.

## 1 Introduction

Globally, the area under tea (*Camellia sinensis* (L.) O. Kuntze (Theaceae)) cultivation has increased and by 2020, global tea production reached 6.269 million tons. This is 1.68 million tons higher compared to 2010. Major tea producing countries include China, India, and Kenya. However, among these, China ranks first with a cultivated area of 3.1669 million hectares (62.1% of the global area) and 2.97 million tons of production (www.weihengag.com; accessed on 16 November 2023). Tea plantations in China extend to Anhui, Hubei, Hunan, Jiangxi, Jiangsu, and Zhejiang provinces (Pan et al., 2022). Green tea is one of the six teas sourced from *C. sinensis*. It is minimally oxidized tea with the least fermentation. The health beneficial components in green tea include polyphenols (flavonoids and phenolic acids), tannins, and saponins. Among these, polyphenols (catechins) are the key bioactive components (Reygaert, 2017). The processing conditions of green tea prevent the oxidization of polyphenols by inactivating the key enzymes, e.g., polyphenol oxidases. However, the contents of these bioactive compounds are affected by growing conditions, genetic background, and most importantly the environmental factors (Astill, Birch, Dacombe, Humphrey and Martin, 2001).

Light and shade are the factors that have been recently explored for their effects on biochemical composition of green tea plants. Particularly, shading with controlled light transmission has been shown to affect the accumulation of the key components in green tea such as catechins, theanine, and caffeine. These changes are due to the increase or decrease in the available carbon and nitrogen sources, thereby enhancing tea taste or quality (Li et al., 2020). This approach of giving shading treatment to tea plants has been previously tested on varieties like matcha and gyokuro. The results of such experiments have shown that shading reduces caffeine content, which is desirable for achieving a smooth taste. Caffeine is a xanthine alkaloid, which is used as a stimulant in medical field and as an analgesic adjuvant (Lin et al., 2023). Earlier studies on the shade treatment to tea plantations have revealed shading can induce photosynthetic (chlorophyll and carotenoid) pigment accumulation (Chen et al., 2021), reduction in catechin and theaflavin content (Yu et al., 2020), decrease in flavonoids (Wang et al., 2012), and changes in the accumulation of other key components including caffeine, tryptophan, glutamine, and glutamate (Elango et al., 2023). The differences in accumulation of the pigments in response to shade are associated with reduced photoinhibition, whereas moderate shading can induce an adaptation response to stress caused by strong light and therefore increases the chlorophyll accumulation (Wang et al., 2019). Whereas, the catechins are biosynthesized by the phenylpropanoid and flavonoid biosynthesis pathways (Deng and Lu, 2017). Earlier research explained that flavonoids are sensitive to light conditions, therefore, total catechin content reduces under shade (Ye et al., 2021). This is because growing tea plants under shade can induce a reduction in the expression of several key genes involved in flavonoid biosynthesis. The reduction in catechins is therefore related to the changes in flavonoid and upstream pathways (Wang et al., 2012). Studies have also elaborated on the regulation of catechin-related genes by the photosynthetic capacity of tea plants (Xiang et al., 2021). Moreover, studies have also indicated that shade treatment also impacts the caffeine metabolism in relation to free amino acids (e.g., theanine). However, the effect of shading on their content is variable in different varieties, indicating the need for exploring variety specific shading effects (Li et al., 2020; Zhang et al., 2022).

Another factor in shading treatment is the color and intensity of the shade. Since natural sunlight consists of a range of wavelengths each of which regulates plant growth differently (Zoratti et al., 2014). Most recently, the focus has been shifted toward the use of green, blue, and red light-emitting diodes under controlled conditions. In case of tea, the use of colored shade-nets has been tested in response to the changes in the metabolite compositions (Jin et al., 2021). Different colored shade nets can reduce the light (as well as UV) intensity as well as change the spectral composition. Such change impacts the biosynthesis of flavonoids, which in turn impacts downstream metabolite composition (Jin et al., 2021; Ye et al., 2021). Some studies have also reported that different colored shading treatments can also alter the endogenous phytohormone content, thus it may lead to changes in the expression of downstream genes and thus, in addition to metabolite changes, influence the plant growth and development (Fang et al., 2022).

Huangjinya, as an excellent green tea variety, has been widely planted in recent years (1.6 million tons, www.weihengag.com; accessed on 16 November 2023). A major observation during the growth period of this variety is its sensitivity towards sunlight. This reduced resistance to sunlight damages the buds and leaves and hence the quality of the green tea (T. Sano, Horie, Matsunaga and Hirono, 2018; Too, Kinyanjui, Wanyoko and Wachira, 2015). Since, Huangjinya is a light-sensitive tea variety (Wang Kairong et al., 2008), continued research on the protection of the buds and leaves from sunlight is required (Shin et al., 2018). Considering the applicability of shading to reduce the sensitivity to sunlight (Elango et al., 2023), it is important to determine how Huangjinya will respond to different shading treatments. Moreover, growing under different colored shading can also reveal the most appropriate shading treatment without affecting the tea quality. Here, we treated Huangjinya tea with different shading nets, i.e., red, yellow, blue, and black (75% and 95% shading rate) to find out the most suitable color of the shading net. We compared the weight of 100 one-bud-three leaves, length of one-bud-three leaves, degree of new shoots burn, and new shoots burn rate (%) of the tea grown under these treatments with the one grown under natural sunlight conditions. Based on the comparison results, we selected Red shading net treatment to understand its effect on global metabolome profile and the associated transcriptome changes in comparison to tea grown with no shading treatment.

## 2 Results

### 2.1 Effect of shading on morphological parameters of tea plants

**FIGURE 1:**
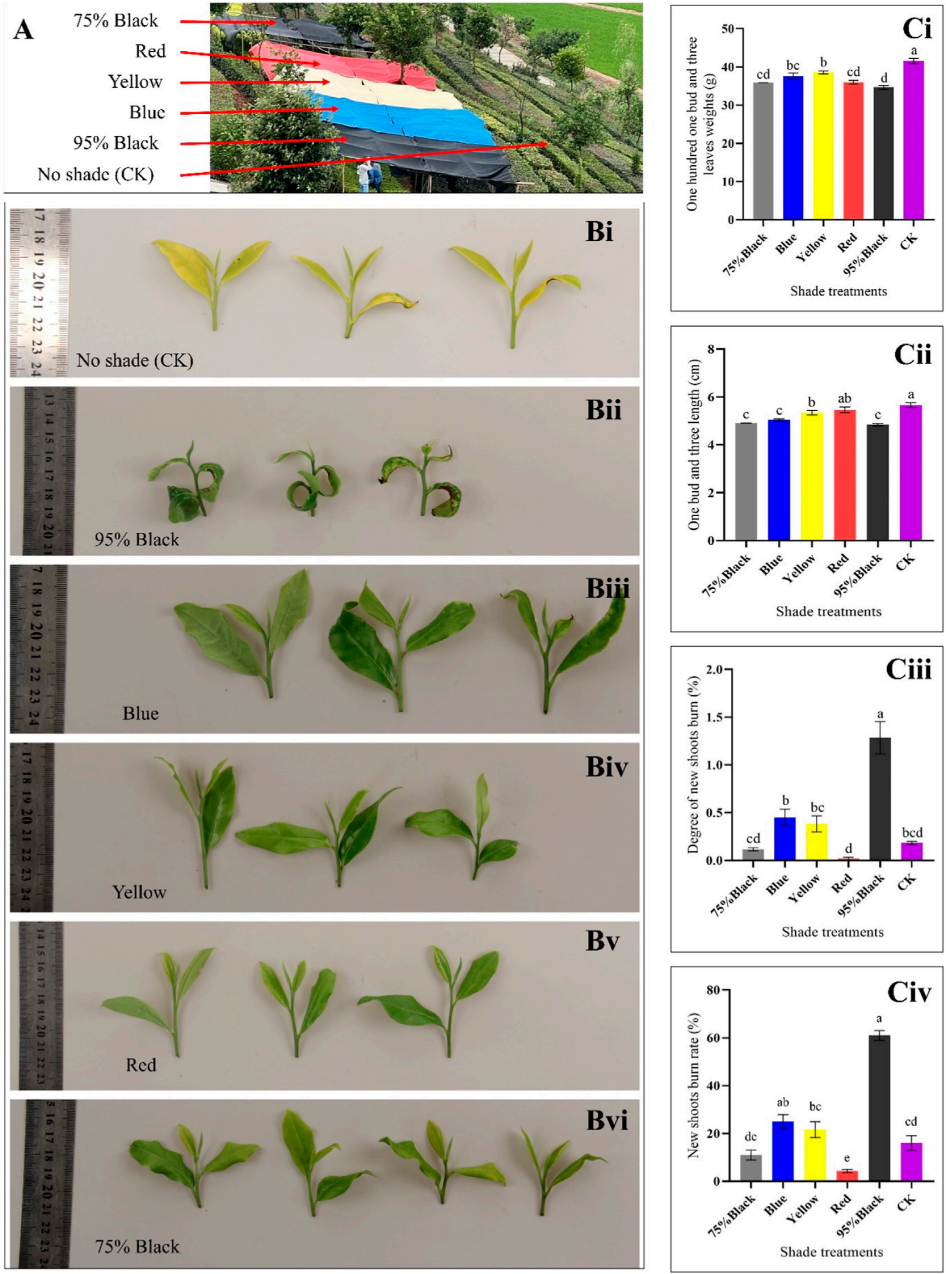
Effect of shading on tea plant. **(A)** Experiment layout. **(B)** Photographs showing the appearance of one-bud-three-leaves samples from the six shading treatments: **(i)** Control (CK), **(ii)** 95% Black, **(iii)** Blue, **(iv)** Yellow, **(v)** Red, and, **(vi)** 75% Black shades. **(Ci)** Weight of 100 One-Bud-Three-Leaves for Six Treatments (grams): Bar chart representing the weight in grams of 100 one-bud-three-leaves samples under different shading conditions. **(Cii)** Length of One-Bud-Three-Leaves (cm): Bar chart illustrating the length in centimeters of one-bud-three-leaves samples for each shading treatment. **(Ciii)** Degree of New Shoots Burn: Bar chart displaying the degree of burning on new tea shoots for the various shading conditions. **(Civ)** New Shoots Burn Rate (%): Bar chart depicting the percentage of new shoots burn rate under different shading conditions, indicating the susceptibility of tender shoots to burning. The letters (a, b, c, d, e) on graph colums are associated with pairwise comparisons. Bars with different letters are significantly different at *p*-value *<* 0.05.

Based on the color and type of shading net, the experiment was divided into six groups, each representing a different shading condition, i.e., red, yellow, blue, black (75% shading rate), black (95% shading rate), and compared to an unshaded control group (CK) (Figure 1A). Shading treatments significantly influenced the morphology of tea buds and leaves (Figure 1B). In CK group, shoot apical leaves and bud were seemingly light green, however, after shading treatment, leaf and bud morphology significantly changed. The weight of 100 one-bud-three-leaves was measured for each shading condition, providing insights into the impact of shading on tea yield. The results indicate that shading significantly influenced the weight of 100 one-bud-three-leaves (H1B3L) in the Huangjinya tea plants. In the absence of shading treatment (CK), the average weight of H1B3L was 41.57 g, which is highest as compared to shading treatments. Notably, the yellow shading treatment resulted in the highest weight, suggesting a potentially positive effect on tea yield when compared to the other colored shading nets. Conversely, red and black (75% and 95%) shading showed a decrease in weight compared to other treatments, indicating a potential negative impact on yield (Figure 1Ci).

Shading conditions significantly influenced the length of one-bud-three-leaves (L1B3L) in Huangjinya tea plants. In the absence of shading treatment (CK), the average (L1B3L) was 5.66 cm. Notably, the Red and yellow shading treatment resulted in the longest leaves among shading treatments, suggesting a potentially positive effect on tea leaf growth and morphology. Conversely, blue and black (75% and 95%) shading showed highest decrease in length compared to other treatments, indicating a potential inhibitory effect on leaf elongation (Figure 1Cii).

The degree of new shoots burn (DNSB) was measured for each shading condition, providing insights into the severity of burning on tender tea shoots. In the absence of shading treatment (CK), the DNSB was 0.18. The results reveal significant variations in the DNSB different shading conditions (Figure 1Ciii). Notably, Red, CK and black (75%) shading exhibited the lowest DNSB, indicating effectiveness in protecting new tea shoots from severe damage. Conversely, 95% black shading resulted in the highest DNSB (1.28), suggesting a higher susceptibility of tender shoots to scorching under this condition.

The rate of new shoots burn (RNSB) was measured for each shading condition, providing insights into the impact of shading on the susceptibility of new tea shoots to burning. The results demonstrate that different shading conditions have a variable impact on the susceptibility of new tea shoots to burning (Figure 1Civ). In the absence of shading treatment (CK), the RNSB was 15.83%. Notably, Red shading exhibited the lowest RNSB (4.17%), indicating that this condition is effective in protecting the tender tea shoots from burning. In contrast, 95% black shading resulted in a substantially higher RNSB, suggesting that this condition is less conducive to shoot protection.

Taken together, the H1B3L, L1B3L, DNSB, and RNSB highlight that Red shading is the most suitable strategy to increase the yield of Huangjinya tea. Based on these results, we further explored the key metabolomic and transcriptomic changes in Huangjinya tea in response to Red shading.

### 2.2 Metabolomics of Huangjinya tea grown under Red shade and no shading

**FIGURE 2:**
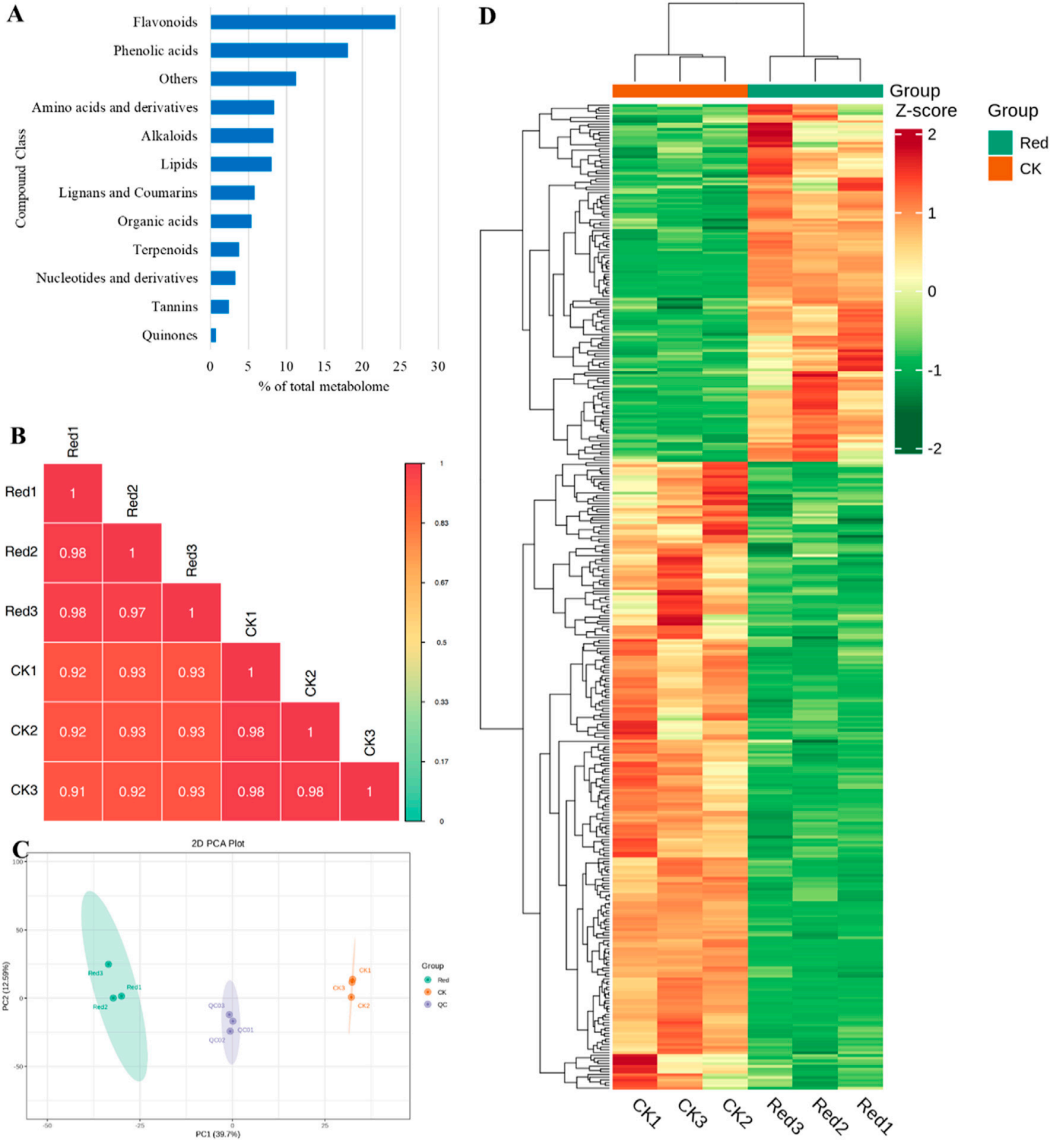
Metabolome analysis of Huangjinya tea grown under Red shade and no shade (CK). **(A)** Percentage of metabolite classes in total number of identified metabolites. **(B)** Pearson’s correlation coefficient, **(C)** Principal component analysis, and **(D)** hierarchical clustering based on relative metabolite intensity mass spectrometry data of each group of samples and quality control samples.

In the current study, a total of 1947 metabolites were identified from CK and one-bud-three-leaves samples from plants given shading treatment with red net (Red). These metabolites belong to 12 major groups of metabolites. Highest number of metabolites were classified as flavonoids (474), followed by phenolic acids (353), and others (219). The two compound classes, i.e., phenolic acids and flavonoids represented 40% of total metabolites (Figure 2A). The correlation between the replicates was ≥0.91 (Figure 2B) and the PCA showed a clear grouping of Red and CK samples (Figure 2C), indicating significant variation in the metabolic profiles between Red and CK. This was further observed in hierarchical clustering analysis (Figure 2D).

### 2.3 Comparative metabolomics profiles of Huangjinya tea grown under Red shade and no shading

**FIGURE 3:**
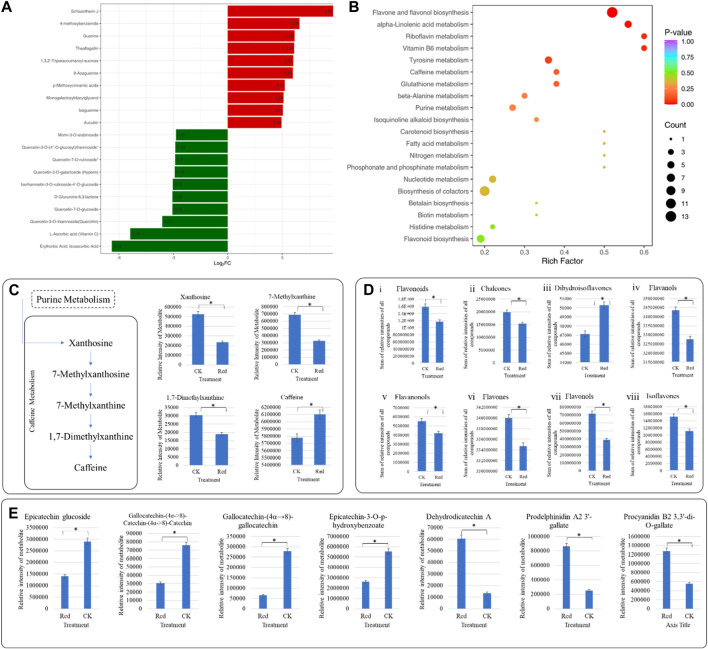
Comparative metabolome profile of Huangjinya tea plants grown under Red shade and no shade (CK) conditions. **(A)** Bar chart of highly up- and down-accumulated metabolites in Huangjinya tea grown under Red shade compared to CK. **(B)** Scatter plot showing top pathways to which DEMs were significantly enriched. The abscissa represents the Rich Factor corresponding to each pathway, the ordinate is the pathway name (sorted according to *p*-value), the color of the point reflects the *p*-value size, and the redder it is, the more significant the enrichment is. The size of the dots represents the number of differentially enriched metabolites. **(C)** Differential caffeine biosynthesis in Huangjinya tea grown under Red shade compared to CK. The bar plots show the metabolites enriched in KEGG pathway Caffeine metabolism. **(D)** Bar plots of flavonoids (and subclasses) accumulated in Huangjinya tea grown under Red shade and no shade conditions. **(E)** Relative content of **(i)** Epicatechin glucoside, **(ii)** Gallocatechin-(4α->8)-Catechin-(4α->8)-Catechin, **(iii)** Gallocatechin-(4α→8)-gallocatechin, **(iv)** Epicatechin-3-O-p-hydroxybenzoate, **(v)** Dehydrodicatechin A, **(vi)** Prodelphinidin A2 3′-gallate, **(vii)** Procyanidin B2 3,3′-di-O-gallate. The bars in **(C–E)**, represent average relative metabolite intensities of metabolites in three replicates.

Out of 1947 annotated metabolites, 361 were differentially accumulated metabolites (DAMs); 230 down-accumulated and 131 up-accumulated (Supplementary Table S2). The top 20 metabolites (up- and down-accumulated) with the highest fold changes are presented in Figure 3A. The top 10 downregulated metabolites in Red samples are representative saccharides (D-glucurono-6,3-lactone), vitamins (L-ascorbic acid (Vitamin C), erythorbic acid; isoascorbic acid) and flavonoids (morin-3-O-arabinoside, quercetin-3-O-(4″-O-glucosyl)rhamnoside*, quercetin-7-O-rutinoside*, quercetin-3-O-galactoside (Hyperin), isorhamnetin-3-O-rutinoside-4′-O-glucoside, quercetin-7-O-glucoside, quercetin-3-O-rhamnoside(quercitrin)). On the other hand, the top 10 upregulated metabolites include alkaloids (4-ethoxybenzamide), lignans and coumarins (schisantherin J), lipids (monogalactosyldiacylglycerol), nucleotides and derivatives (guanine, 8-azaguanine, isoguanine), others (p-methoxycinnamic acids), phenolic acids (1,3,2′-triparacoumaroyl-sucrose), tannins (theaflagallin), and terpenoids (aucubin).

KEGG pathway enrichment analysis was performed using DAMs. Notably, flavone and flavonol biosynthesis, alpha-linolenic acid metabolism, riboflavin metabolism, vitamin B6 metabolism, tyrosine metabolism, and caffeine metabolism had the lowest *p-values* (Figure 3B)*.* Using the KEGG annotation information of DAMs identified according to the screening criteria (VIP > 1; |Log2 FC| ≥ 1.0), five classes of significantly enriched metabolites were selected, and cluster analysis was performed (Supplementary Figure S1).

Considering the differential accumulation patterns of different compound classes, we noticed that most of the alkaloids (alkaloids and plumeranes) were down-accumulated in Red. However, two of the three phenolamines (caffeoylagmatine and N-feruloylhomoagmatine) and the only DAM classified as pyroole alkaloid (methyl L-pyroglutamate) were up-accumulated in Red compared to CK. In case of amino acids and derivatives, all metabolites (except L-homomethionine, L-phenylalanyl-L-phenylalanine, and val-trp) were down-accumulated in response to growing in Red shade. A similar response was recorded for almost all compounds classified as flavonoids. Contrary to these compounds, a notable increase was observed in compounds classified as lipids (free fatty acids) and organic acids. These organic acids include malonic acid, methanesulfonic acid, shikimic acid, jasmonic acid, methyl jasmonate, 6-hydroxyhexanoic acid, tianshic acid, and urocanic acid. Phenolic acids, tannins, and terpenoids exhibited variable patterns (Supplementary Table S2).

In current study, 15 metabolites classified as amino acids and derivatives were differentially accumulated; 3 up- and 12 down-accumulated (Supplementary Table S2). N-Methyl-α-aminoisobutyric acid is a derivative and was down-accumulated in Red samples compared to CK (Supplementary Table S2). Among others, theanine was non-significantly regulated. Our results suggest that though not differentially accumulated, caffeine content was higher in Red compared to CK. This increased accumulation can be due to the increased conversion of xanthosine and 7-methylxanthin to caffeine in response to Red shade (Figure 3C).

In case of flavonoids, we observed a reduction in total flavonoid content in Red compared to CK (Figure 3Di). This reduction could be due to chalcones, flavanols, flavanonols, flavones, flavonols, and isoflavones (Figures 3Dii–vii). This reduced flavonoid content is consistent with the observed changes in the relative intensities of catechin and epicatechin and/or related metabolites (Figure 3E). Moreover, myricetin, luteolin, quercetin, kaempferol, rutin, and naringenin content were also decreased in Red samples (Supplementary Table S2). This could explain the changes in the leaf color observed in the samples grown under Red shade as compared to CK (Figure 1B). Whereas, the dihydroisoflavones had relatively higher content in Red compared to CK. Possibly, this is the reason for increased content of prodelphinidin A2 3′-gallate, and procyanidin B2 3,3′-di-O-gallate. These are the proanthocyanidins found in tea, and their presence can influence the overall sensory experience of tea (Figure 3Diii; Supplementary Table S2).

The tyrosine metabolism pathway had five significantly enriched metabolites. Of which, dopamine and L-tyramine were down-accumulated in response to growing tea plants under Red shade. On the contrary, 3,4-d 2,5-dihydroxybenzoic acid; gentisic acid, and homogentisic acid had higher relative intensities in Red compared to CK (Table 1). Interestingly, the Red shade resulted in the up-accumulation of all the metabolites enriched in linoleic acid and alpha-linoleic acid metabolism (Table 1). Two of the three vitamin B metabolism pathway related metabolites, i.e., pyridoxine and pyridoxal, were up-accumulated in response to growing tea under Red shade compared to CK. A notable observation was the up-accumulation of ribulose-5-phosphate and down-accumulation of flavin single nucleotide and lumichrome; which were enriched in riboflavin metabolism pathway (Table 1).

**TABLE 1:** List of differentially accumulated metabolites in tyrosine, linoleic acid, alpha-linolenic acid, vitamin B6, and riboflavin metabolism pathways.

Index	Class I	CK	Red	VIP	*p*-value	Log2FC
Tyrosine metabolism						
3,4-Dihydroxybenzeneacetic acid*	Phenolic acids	154417	389739	1.31	0.01	1.34
2,5-Dihydroxybenzoic acid; Gentisic Acid*	Phenolic acids	52127	108596	1.36	0.00	1.06
Dopamine	Alkaloids	1003840	293651	1.41	0.00	−1.77
L-Tyramine	Alkaloids	134,234	56293	1.11	0.03	−1.25
Homogentisic acid*	Phenolic acids	88,293	300286	1.23	0.01	1.77
Linoleic acid metabolism						
7S,8S-DiHODE; (9Z,12Z)-(7S,8S)-Dihydroxyoctadeca-9,12-dienoic acid*	Lipids	16426	34147	1.39	0.00	1.06
13S-Hydroperoxy-9Z,11E-octadecadienoic acid	Lipids	9177	46563	1.00	0.19	2.34
alpha-Linolenic acid metabolism						
2-Dodecenedioic acid	Lipids	1192934	6008854	1.42	0.00	2.33
Jasmonic acid	Organic acids	808488	1684853	1.38	0.02	1.06
Methyl jasmonate	Organic acids	162508	326701	1.30	0.00	1.01
9-Hydroxy-12-oxo-10(E),15(Z)-octadecadienoic acid	Lipids	3242	7763	1.42	0.00	1.26
Vitamin B6 metabolism						
L-Glutamine	Amino acids and derivatives	4944196	1853799	1.41	0.00	−1.42
Pyridoxine	Others	142053	430362	1.41	0.01	1.60
Pyridoxal	Others	18867	83287	1.41	0.00	2.14
Riboflavin metabolism						
Ribulose-5-phosphate	Others	212119	766609	1.37	0.00	1.85
Flavin Single Nucleotide(FMN)	Nucleotides and derivatives	3489	0	1.31	0.07	−8.59
Lumichrome	Alkaloids	36143	12122	1.26	0.09	−1.58

Taken together, these observations signify that Red shade grown tea is richer in lipids and organic acids and have reduced flavonoid, alkaloids, and amino acids and derivatives. Moreover, Red shade growing causes reduced catechin, epicatechin, dopamine, L-tyramine, and others and increased caffeine content.

### 2.4 Transcriptome sequencing of Huangjinya tea grown under Red shade and no shade conditions

**FIGURE 4:**
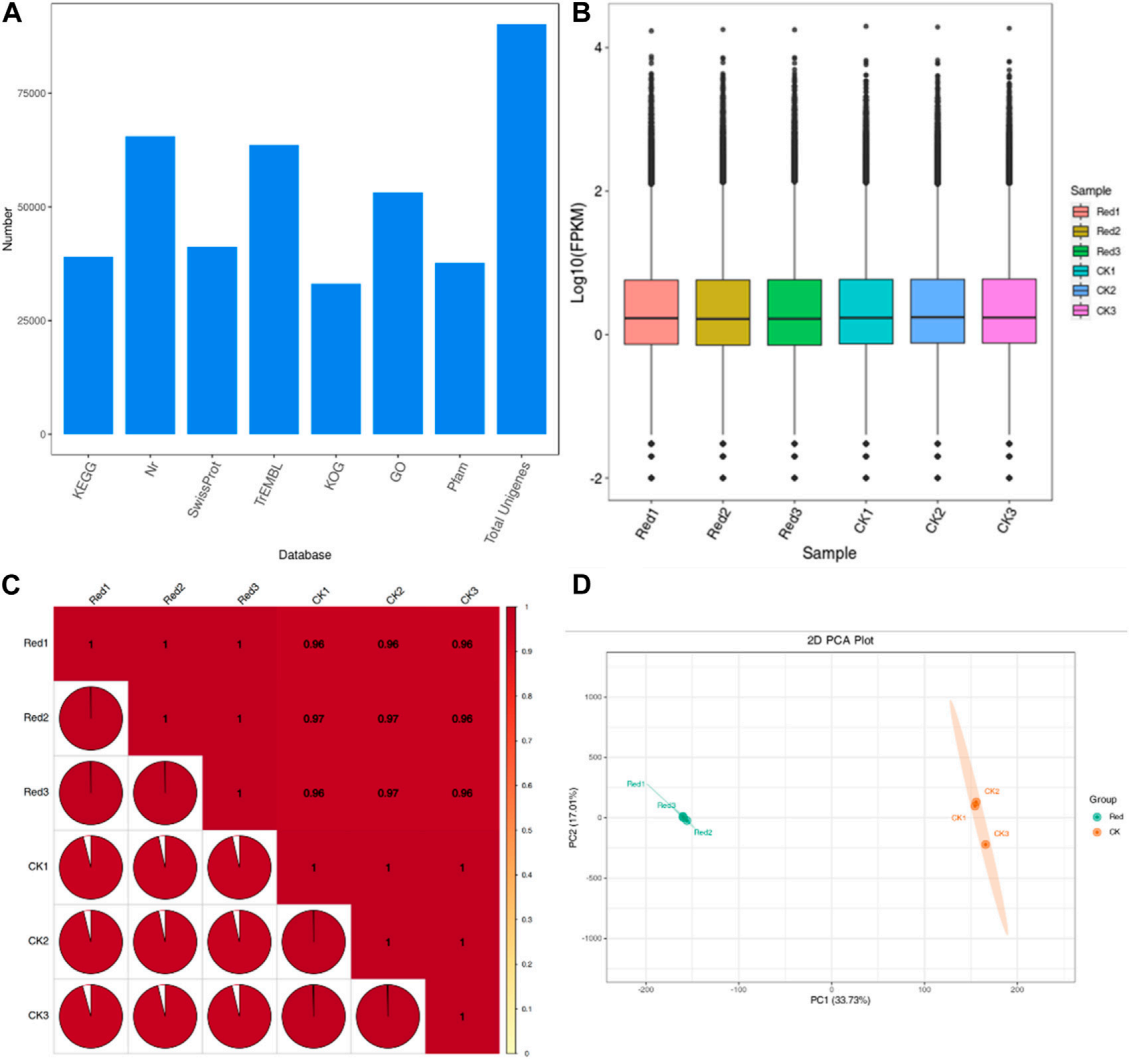
Transcriptome sequencing of Huangjinya tea grown under Red shade and no shade (CK) conditions. **(A)** Annotation of unigenes in different databases. **(B)** Overall distribution of gene expression (FPKM) in six samples. **(C)** Pearson’s Correlation, and **(D)** Principal component analysis of six Huangjinya tea samples based on FPKM values. The numbers with Red and CK indicate replicates.

The sequencing analysis of six libraries produced a total of 40.96 Gb clean data with an average of 6 Gb per sample. The Q30 base percentage, average sequencing error rate, and GC content were ≥94%, 0.02%, and 45%, respectively. Out of the 169,749 transcripts, 90,126 were successfully assembled into unigenes (Supplementary Table S3) with N50 and N90 values of 2011 and 614, respectively. The unigenes could be annotated in various databases: KOG (36.7%), Pfam (41.83%), KEGG (43.27%), SwissProt (45.76%), GO (58.99%), TrEMBL (70.59), and NR (72.67%) (Figure 4A). Overall gene expression of the six libraries is presented in Figure 4B. The PCC between the replicates was very high (0.96–1) (Figure 4C). Moreover, the PCA showed that replicates of the CK and Red tended to group (Figure 4D). Overall, these results indicate that sampling as well as sequencing data is of good quality.

### 2.5 Differential gene expression between Huangjinya tea grown under Red shade and no shade conditions

**FIGURE 5:**
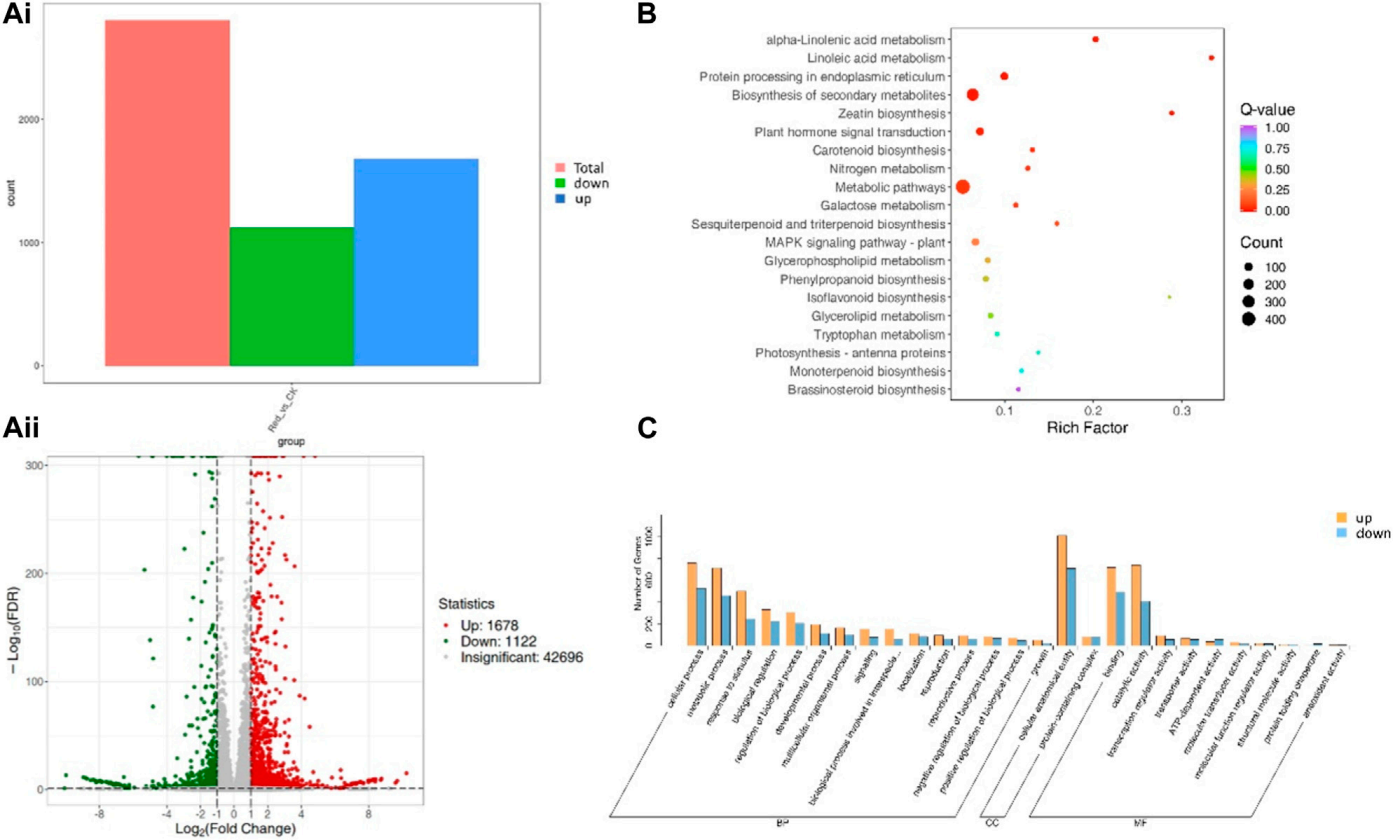
Differential gene expression between Huangjinya tea grown under Red shade and no shade (CK). **(Ai)** Statistics of DEGs, **(Aii)** differential gene volcano plot. The abscissa represents the fold change of gene expression, and the ordinate represents the significance level of differential genes. Red dots represent upregulated differential genes, green dots represent downregulated differential genes, and gray dots represent non-differentially expressed genes. **(B)** KEGG enrichment scatter plot. The ordinate represents the KEGG pathway. The abscissa represents Rich factor. The larger the Rich factor, the greater the degree of enrichment. The larger the point, the greater the number of differential genes enriched in the pathway. The redder the color of the point, the more significant the enrichment is. **(C)** Differential gene secondary entry classification based on GO database.

A total of 2,800 genes were differentially expressed between Red and CK; 1,122 and 1,678 genes were down- and upregulated in Red compared to CK (Figure 5A). KEGG pathway enrichment analysis identified 129 pathways to which the DEGs were significantly enriched. Notably, the DEGs were enriched in alpha-linolenic acid metabolism, linoleic acid metabolism, carotenoid biosynthesis, flavone and flavonol biosynthesis, flavonoid biosynthesis, plant hormone signal transduction, and phenylpropanoid biosynthesis (Figure 5B). It is understandable from the enrichment analysis that several pathways related to amino acid metabolism were enriched. This is consistent with the metabolome profile. The GO classification showed that highest number of genes were associated with cellular anatomical entity, cellular process, binding, catalytic activity, metabolic process, and response to stimulus (Figure 5C).

The highly upregulated genes in response to Red shade treatment included *brassinosteroid insensitive 1-associated receptor kinase 1-like* (*Cluster-13486.2*), *UDP-glucose/iron transport system ATP-binding protein* (*Cluster-41545.2*), *protein transport protein SEC24* (*Cluster-40691.2*), and *acetyl-CoA acyltransferase 1* (*Cluster-51277.4*). These expression changes highlight potential activation of genes enriched in signaling (phytohormone and MAPK), protein processing, and fatty acid metabolism pathways in response to Red shade. On the contrary, the most downregulated genes in Red shade grown tea were *nucleoprotein TPR* (*Cluster-48385.6*), *type III protein arginine methyltransferase* (*Cluster-46255.1*), *deoxynucleoside triphosphate triphohydrolase SAMHD1* (*Cluster-53979.6*), and *E3 ubiquitin-protein ligase HOS1* (*Cluster-53716.1*). These expressions indicate nucleocytoplasmic transport, ribosome, and related pathways are affected if the tea is grown under Red shade compared to CK (Supplementary Table S4).

#### 2.5.1 Expression changes in lipid metabolism and associated pathways are consistent with metabolome profile

**FIGURE 6:**
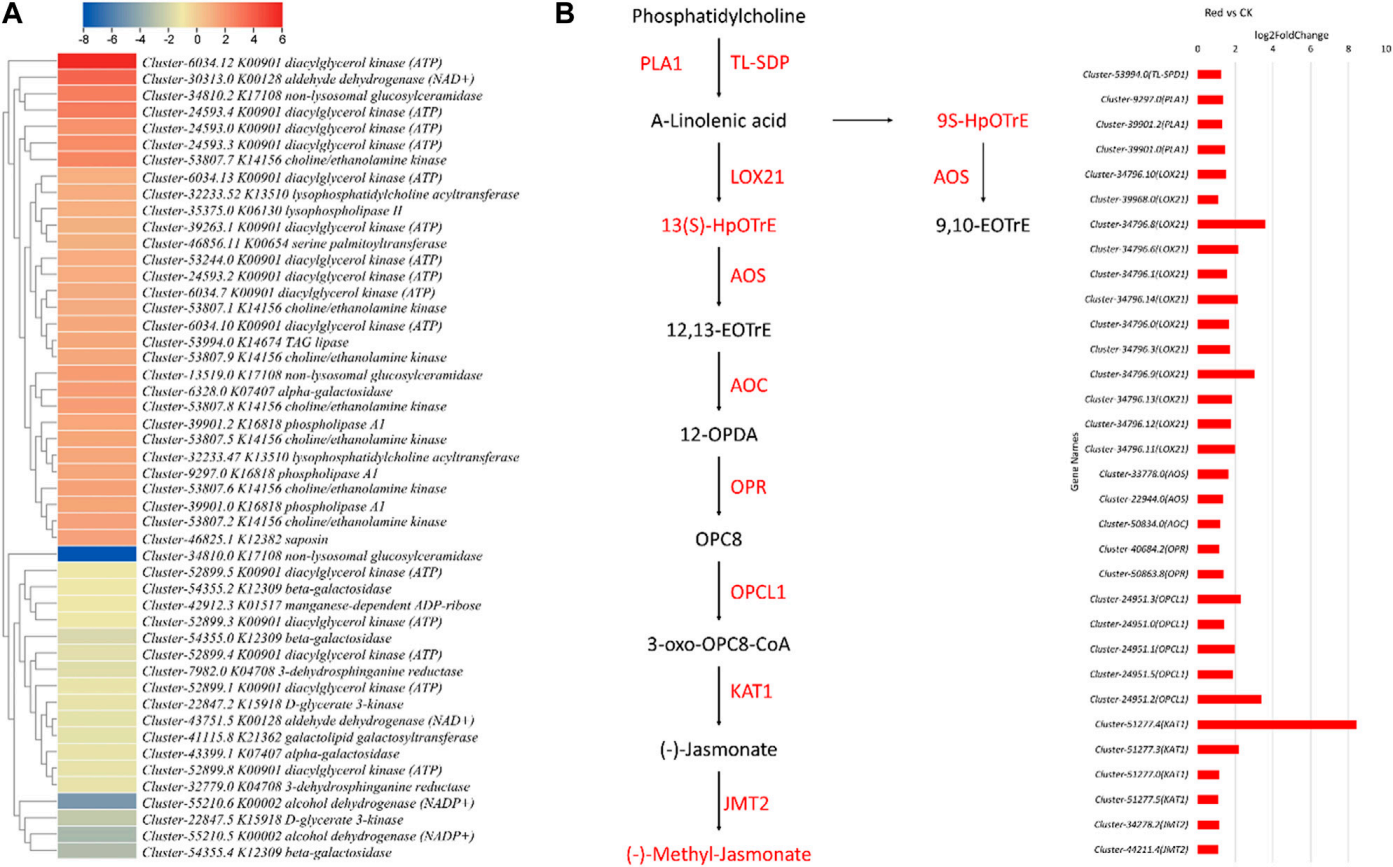
Expression changes in lipid metabolism (and related pathways), alpha-linolenic acid, and Jasmonate biosynthesis pathways. **(A)** Heatmap of log2 foldchange values of genes enriched in all lipid-related pathways. **(B)** Alpha-linolenic acid and jasmonate biosynthesis pathway. The red color represents the upregulation of genes in Red samples compared to no shading (CK). Names in boxes represent metabolites and names along arrows represent genes. Red: tea plants under red color shading. The bar graph shows the log2 foldchange of genes shown in the pathway. The red color represents the upregulation of genes in Red samples.

The metabolome profile of the Red and CK showed increased lipid contents in the Red. Therefore, we searched for the associated genes and their expression trends in response to Red shade growth. Six lipid-related pathways were significantly enriched, i.e., glycerophospholipid metabolism (and also the - ganglio series and - globo and isoglobo series), glycerolipid metabolism, sphingolipid metabolism, and ether lipid metabolism. A total of 49 DEGs annotated as 17 KEGG ontology terms were differentially expressed between Red and CK. This indicates multiple transcripts of the same gene were detected in our transcriptome sequencing. Thirty-one of these were upregulated in response to Red shade compared to CK. *Triacylglycerol lipase* (*TL-SDP*; *Cluster-53994.0*), *phospholipase A1* (*PLA1*; *Clusters 9,297.0, 39,901.2, 39,901.0*) involved in lipid metabolism, alpha-linolenic acid metabolism, and various KEGG pathways was significantly upregulated, suggesting an increase in lipid metabolism in response to Red shading. *Lipoxygenase gene* (*LOX21*; *Clusters 34,796.10, 39,968.0, 34,796.8, 34,796.6, 34,796.1, 34,796.14, 34,796.0, 34,796.3, 34,796.9, 34,796.13, 34,796.12, 34,796.11*) responsible for fatty acid oxidation and linoleic acid metabolism was significantly upregulated (Figure 6A). On the contrary, only a few were downregulated. These downregulated included *alcohol dehydrogenase (NADP+)*, *beta-galatosidase* (*Cluster-54355.2*, *Cluster-54355.0*, and *Cluster-54355.4*), *manganese-dependent ADP-ribose/CDP-alcohol diphosphatase* (*Cluster-42912.3*), *D-glycerate 3-kinase* (*Cluster-22847.2* and *Cluster-22847.5*), *non-lysosomal glucosylceramidase* (*Cluster-34810.0*), *3-dehydrosphinganine reductase* (*Cluster-32779.0* and *Cluster-7982.0*). Overall, these observations are consistent with the metabolome profile of the Red shade grown Huangjinya tea samples. These results clearly indicate that Red shade triggers higher expression of several genes associated with lipid biosynthesis, and therefore increases bud lipid contents.

Furthermore, the upregulation of *allene oxide synthase* (*AOS*; *Cluster-33778.0*) and *allene oxide cyclase* (*AOC*; *Cluster-50834.0*) suggests enhanced jasmonic acid production. Notably, *Cluster-40684.2* (*OPR*), responsible for converting 12-oxo-phytodienoic acid to jasmonic acid, showed upregulation in the Red. This suggests increased jasmonic acid biosynthesis, which is consistent with the metabolome profile of samples grown under Red shade. Similarly, *Cluster-24951.3* (*OPCL1*), involved in OPC-8:0 CoA ligase 1 activity, exhibited upregulation, suggesting its involvement in the jasmonic acid precursor synthesis. *Cluster-51277.4* (*KAT1*), a *3-ketoacyl CoA thiolase*, was upregulated, which could influence lipid metabolism. Lastly, *JMT2*, a *jasmonate O-methyltransferase* (*Cluster-34278.2* and *Cluster-44211.4*), was upregulated, potentially affecting jasmonic acid signaling. These results are in accordance with metabolome results for an up-accumulation of jasmonic acid and methyl jasmonate (Figure 6B; Supplementary Table S2). Overall, the data indicate a significant impact of Red shading on the jasmonic acid-related pathways and lipid metabolism.

#### 2.5.2 Expression changes in phytohormone signaling pathways

**FIGURE 7:**
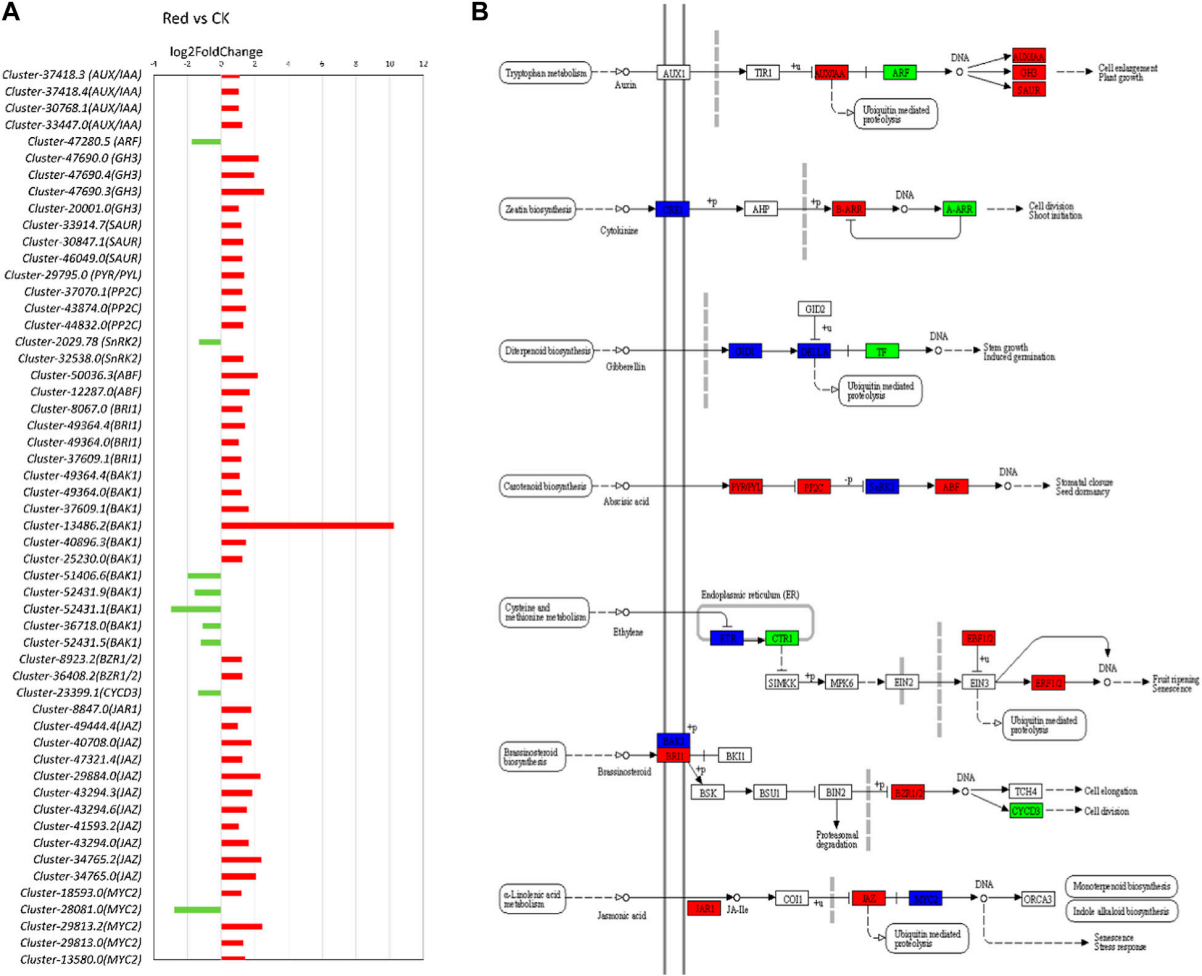
Plant hormone signal transduction pathway. **(A)** Fold change expression of genes involved in plant hormone signal transduction pathway. The red color represents the upregulation of genes in Red samples and the green color represents downregulation of genes in Red samples. **(B)** Pathway representation of plant hormone signal transduction pathway genes. The red color represents the upregulation of genes in Red samples. Names in boxes represent genes. Red: tea plants under red color shading. CK: no shading.

In the transcriptome analysis comparing Red and CK samples, several key insights into the regulation of phytohormone signaling pathways emerge. Notably, we observe significant changes in the expression of genes associated with various phytohormones (Figure 7). Regarding auxin signaling, we noticed that *AUX1* and *TIR1* were not differentially expressed. *AUX1* and *TIR1* are involved in auxin transport and perception, respectively. It suggests that the capacity for auxin uptake and perception remains relatively stable in the cells or tissues analyzed. Upregulation of *AUX/IAA* transcripts (*Cluster-37418.3, Cluster-37418.4, Cluster-30768.1,* and *Cluster-33447.0*) would lead to an increased abundance of Aux/IAA repressor proteins. This can result in stronger repression of auxin-responsive gene, potentially inhibiting certain auxin-mediated responses. Downregulation of ARF gene (*Cluster-47280.5*) might result in decreased levels of ARF transcription factors, leading to a reduced capacity to activate the transcription of auxin-responsive genes, further impairing the ability of cells to respond to auxin signals. *GH3* (*Cluster-47690.0, Cluster-47690.4 Cluster-47690.3, Cluster-20001.0*) and *SAUR* (*Cluster-33914.7, Cluster-30847.1, Cluster-46049.0*) were upregulated. Considering their role in auxin homeostasis and the modulation of auxin responses, their upregulation indicates an attempt to regulate auxin levels and responses thereof in response to growing under Red shade. The observations that 2-oxindole-3-acetic acid was down-accumulated in Red shade grown plants (Supplementary Table S2), suggests that there is less available auxin for cellular processes.

In cytokinin signaling, *CRE1* is a cytokinin receptor. Transcripts related to this gene were either upregulated (*Cluster-49104.4, Cluster-49104.8*) or downregulated (*Cluster-49104.0*). *AHP* (*Arabidopsis Histidine Phosphotransfer Protein*) is involved in transmitting cytokinin signals downstream. Non-significant regulation of this gene might mean that the level of *AHP* is not changing significantly in response to cytokinin signaling. Contrarily, B-ARRs (Type-B Response Regulators) transcripts (*Cluster-9972.4, Cluster-44927.8*) were upregulated. It implies that the plant is preparing for enhanced transcription of cytokinin-responsive genes. Similarly, A-ARRs (Type-A Response Regulators; negative regulators of cytokinin signaling) transcript (*Cluster-22635.1*) was downregulated (Figure 7). Although, in metabolome analysis, cytokinin-related metabolites were not differentially accumulated (Supplementary Table S2), the expression of these genes indicates that the Red shade is potentially reducing plants’ ability to inhibit cytokinin signaling. This would enhance the response to cytokinins.

Several transcripts related to gibberellic acid, i.e., *GID1* and *DELLA* showed variable expression patterns, suggesting a balanced response (Figure 7; Supplementary Table S4). A similar but complex response was observed for ABA signaling related genes. Notably, the upregulation of *PYR/PYL* (*Cluster-29795.0*) and *ABF* (*Cluster-50036.3, Cluster-12287.0*) transcripts indicates an increased capacity for ABA perception and ABA-responsive gene expression. However, the upregulation of *PP2C* (*Cluster-37070.1, Cluster-43874.0, Cluster-44832.0*) and the mixed response in *SnRK2* transcripts (Upregulation: *Cluster-2029.78*, Downregulation: *Cluster-32538.0*) suggest that there are regulatory mechanisms in operation to balance and fine-tune the ABA signaling pathway. This is consistent with the observation that abscisic acid D-glucopyranosyl ester (ABA-GE; a natural plant hormone derived from ABA) was up-accumulated in Red shade grown plants (Supplementary Table S2). ABA-GE is a conjugate of ABA and glucose, which can convert back to ABA, helping plants control ABA levels for stress responses and growth regulation. Similar to GA and ABA, the ethylene pathway exhibited complex changes. For example, some genes such as *Cluster-32377.0* (*EIN3-binding F-box protein*) associated with ethylene receptors are downregulated, while others are upregulated. The genes enriched in brassinosteroid signaling pathway also showed variable expression patterns. Notably, *BAK1* (*BRI1-associated receptor kinase*) transcripts were either upregulated (*Cluster-44900.0, Cluster-13486.3, Cluster-13486.1, Cluster-13486.2, Cluster-40896.3, Cluster-25230.0*) or downregulated (*Cluster-51406.6, Cluster-52431.9, Cluster-52431.1, Cluster-36718.0, Cluster-52431.5*) and *BRI1* (*BRASSINOSTEROID INSENSITIVE 1*) transcripts (*Cluster-8067.0, Cluster-49364.4, Cluster-49364.0, Cluster-37609.1*) were upregulated. Meanwhile, *BZR1* (*Brassinosteroid resistant ½*) transcript (*Cluster-8923.2, Cluster-36408.2*) were upregulated, suggesting downstream signaling components are being activated. However, downstream genes are either non-significantly regulated or downregulated (*Cyclin D3* or *CYCD3*: *Cluster-23399.1*).

As presented in the above section, we observed both differential accumulation as well as expression related to JA biosynthesis. In this regard, several genes associated with the JA signaling pathway were upregulated (Figure 7). Among these genes, *jasmonoyl--L-amino acid synthetase JAR4* (*Cluster-8847.0*), and *TIFY 10A* (*Cluster-49444.4*) had notably higher expressions in Red shade grown tea plants. *JAR4* plays a crucial role in JA biosynthesis, whereas *TIFY* is associated with the *jasmonate ZIM domain-containing protein* (*JAZ*). *JAZ* is a major player in the jasmonic acid signaling pathway. Additionally, several genes encoding different members of the TIFY family, such as *TIFY 6B* and *TIFY 9*, were upregulated in response to Red shading. These results suggest that Red shading leads to the activation of the JA signaling pathway. Considering the role of JA in stomata functioning, we also noted that 27 or 33 DEGs with stomata related GO terms showed increased expression in response to growing the plants under Red shade nets (Supplementary Table S4). Thus a probable link between JA and better performance of tea plants under Red shade nets can be linked with improved stomata functioning.

#### 2.5.3 Expression changes in phenylpropanoid and flavonoid biosynthesis pathways

**FIGURE 8:**
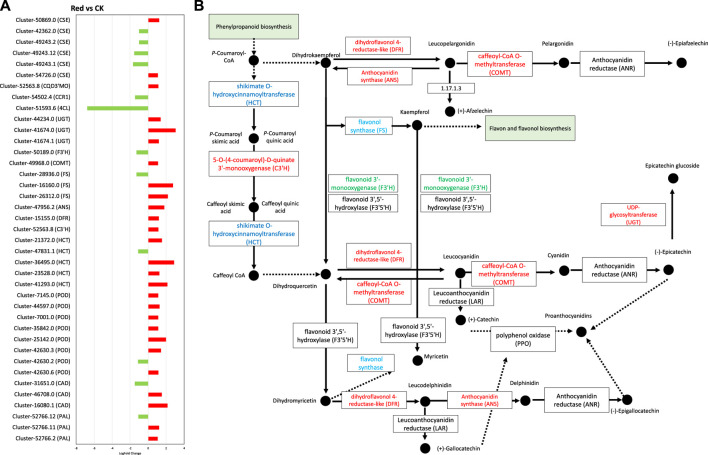
Phenylpropanoid and flavonoid biosynthesis pathway. **(A)** Fold change expression of genes involved in Phenylpropanoid and flavonoid biosynthesis pathway. The red color represents the upregulation of genes in Red samples and the green color represents downregulation of genes in Red samples. **(B)** Pathway representation of Phenylpropanoid and flavonoid biosynthesis pathway genes. The red color represents the upregulation of genes in Red samples. The blue color represents some transcripts of the gene are upregulated while some are downregulated. Names in boxes represent genes. Red: tea plants under red color shading. CK: no shading.

Since phenylpropanoid biosynthesis pathway was significantly enriched (Figure 8). We explored expression changes in related genes. From the 32 DEGs enriched in this pathway (Supplementary Table S4), 22 were upregulated. However, two specific genes, i.e., *4-coumarate-CoA ligase* (*4CL*) and *cinnamyl-alcohol dehydrogenase* (*CAD*) were downregulated. This indicates that in response to Red shade growing, the conversion of cinnamic acid, p-coumaric acid, caffeic acid, ferulic acid, and sinapic acid to cinnamaldehyde, p-coumaraldehyde, caffeoyl-aldehyde, coniferyl-aldehyde, and sinapaldehyde, respectively, was reduced. However, the genes enriched in the part of pathway that leads conversion of cainnamoyl-CoA to caffeoyl-Coa, then to feruloyl-CoA and sinapyl-CoA were mostly upregulated. These include 5*-O-(4-coumaroyl)-D-quinate 3′-monooxygenase* (*C3′H*), *caffeoyl-CoA O-methyltransferase* (*COMT*), and *coniferyl-aldehyde dehydrogenase* (*REF1*) (Figure 8). This indicates Red shade influences the phenylpropanoid biosynthesis pathway in such a way that resources are directed less towards flavonoid biosynthesis as noticed in metabolome biosynthesis.

In flavonoid biosynthesis pathway (Figures 8A, B), several key genes and clusters exhibited significant changes in expression levels. *Cluster-52766.2*, *Cluster-52766.11*, and *Cluster-52766.12*, which correspond to *phenylalanine ammonia-lyase* (*PAL*), showed upregulation in the Red group. PAL is a crucial enzyme in the phenylpropanoid pathway, which is involved in the synthesis of phenolic compounds. These findings suggest that the Red shading treatment enhances the production of phenolic compounds in tea leaves. *Cluster-41293.0*, *Cluster-23528.0*, *Cluster-36495.0*, and *Cluster-21372.0*, which are associated with *shikimate O-hydroxycinnamoyltransferase* (*HCT*), also exhibited upregulation in the Red group. *HCT* is involved in the biosynthesis of lignin precursors and other secondary metabolites. In contrast, *Cluster-47831.1*, associated with *HCT*, showed downregulation in the Red group, indicating a complex regulation of this gene family in response to shading. Additionally, *Cluster-52563.8*, which corresponds to *C3′H*, was upregulated in the Red group. *C3′H* plays a role in the biosynthesis of chlorogenic acid, a major phenolic compound in tea. Furthermore, *Cluster-15155.0*, associated with *dihydroflavonol 4-reductase* (*DFR*), showed upregulation in the Red group. *DFR* is involved in anthocyanin biosynthesis, contributing to the color of tea leaves. Cluster-47956.2, associated with *anthocyanidin synthase* (*ANS*), exhibited upregulation in the Red group, indicating an increased synthesis of anthocyanin compounds. Clusters related to *flavonol synthase* (*FS*), including *Cluster-26312.0, Cluster-16160.0,* and *Cluster-28936.0*, showed a mix of upregulation and downregulation. *FS* is involved in the biosynthesis of flavonols, another group of flavonoids, with distinct functions in plants. *Cluster-49968.0*, associated with *COMT*, showed upregulation in the Red group, suggesting an increased methylation of phenolic compounds. Conversely, *Cluster-50189.0*, corresponding to *flavonoid 3′-monooxygenase* (*F3′H*), was downregulated in the Red group. *F3′H* is involved in the biosynthesis of flavonoids. Clusters associated with *UDP-glycosyltransferase* (*UGT*) exhibited upregulation (*Cluster-41674.1, Cluster-41674.0*, *Cluster-44234.0*) in the Red group. *UGT* is involved in the glycosylation of flavonoids, affecting their bioavailability and stability (Figure 8). Collectively, these findings suggest that subjecting tea leaves to Red shading treatment results in intricate modulation of genes associated with the synthesis of diverse phenolic compounds. This modulation has the potential to impact the flavor, color, and potential health benefits of tea (Zeng et al., 2019; Zhao et al., 2023).

Interestingly, *anthocyanidin reductase* (*ANR*) and *leucoanthocyanidin reductase* (*LAR*), critical enzymes for polymerization of catechin and epicatechin were not differentially regulated. However, *ANS* and *UGT* were upregulated, suggesting a potential trend of polymerization or condensation of simple molecules like catechin or pelargonidin into larger molecules like glucoside or proanthocyanidins.

In addition to the above mentioned pathways, we observed that 19 amino acid metabolism related pathways were significantly enriched. Among these, taurine and hypotaurine metabolism, valine, leucine, and isoleucine biosynthesis, tyrosine metabolism, histidine metabolism, and cysteine and methionine metabolism had a relatively higher number of downregulated transcripts in Red treatment compared to CK. These changes are consistent with the observed different accumulation of several amino acids and derivatives in Red (Supplementary Table S5).

### 2.6 Validation of differentially expressed genes

**FIGURE 9:**
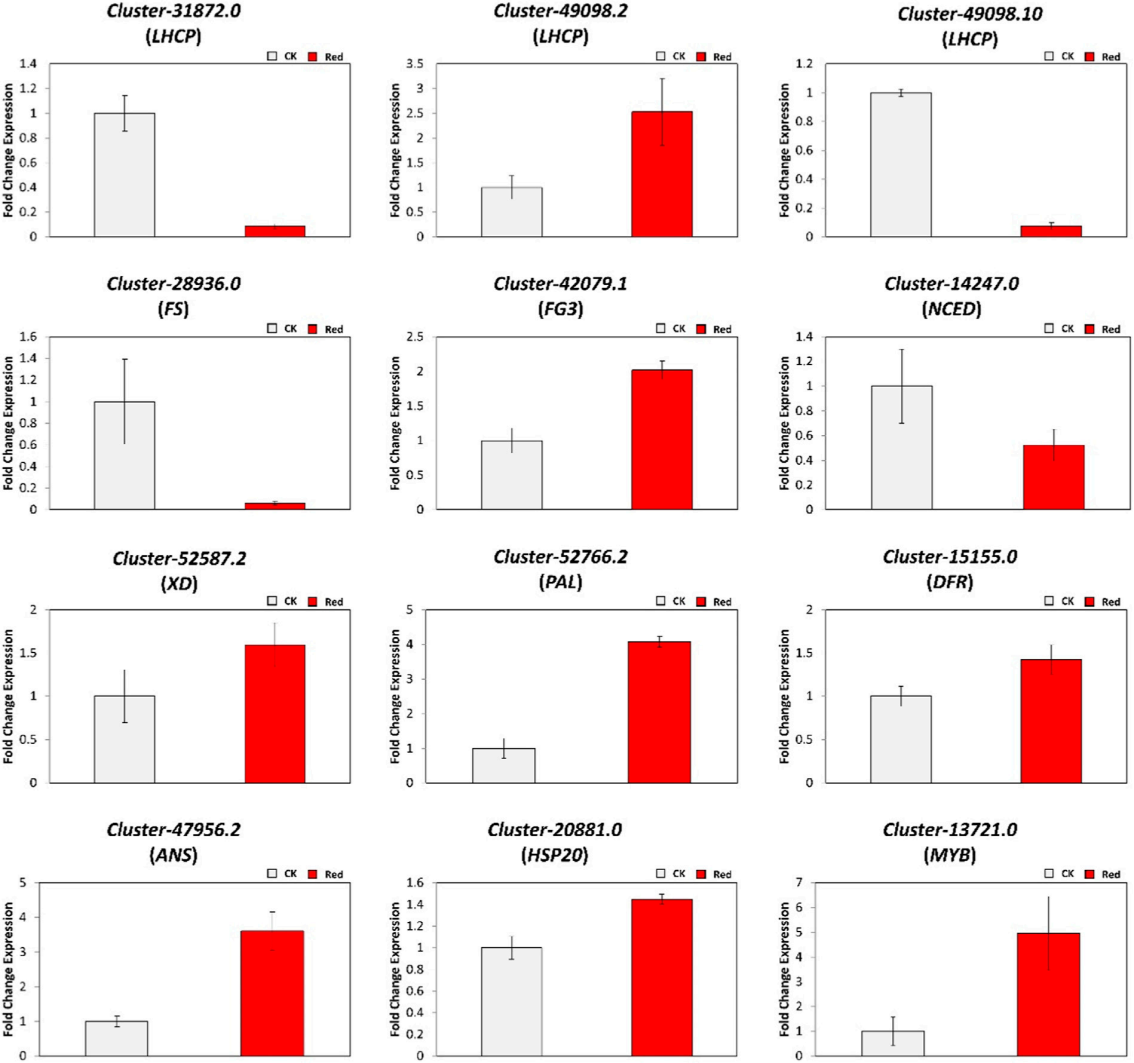
Relative expression levels of DEGs identified in the comparison of CK vs. Red group using qRT-PCR.

To verify the validity of the RNA sequencing results, we randomly selected 12 differentially expressed genes (DEGs) for qRT-PCR analysis. These DEGs were related to *chlorophyll a-b binding protein* (*LHCP*: *Cluster-31872.0, Cluster-49098.2,* and *Cluster-49098.10*), phenylpropanoid and flavonoid pathway (*Cluster-28936.0 (FS), Cluster-42079.1 (FG3), Cluster-14247.0 (NCED), Cluster-52587.2 (XD), Cluster-52766.2 (PAL), Cluster-15155.0 (DFR),* and *Cluster-47956.2 (ANS)*), and transcription factors (*Cluster-20881.0 (HSP20), Cluster-13721.0 (MYB),* and *Cluster-42072.10 (WRKY33*). As shown in Figure 9, qRT-PCR data trends were similar to tramscriptome data in response to Red shading. These results confirmed the reliability of the RNA sequencing results and reflected the transcriptome changes observed.

## 3 Discussion

Huangjinya tea is a new variety and is considered difficult to cultivate owing to its light sensitivity. Therefore, it requires relatively higher cost for management and production. However, knowledge of how the effect of different colored shading nets can alter its metabolome as well as related differential transcriptome changes is almost non-existent. This knowledge is essential to understand the key pathways that are responsive to shading nets. In this study, we have tested the morphophysiological, metabolomic and transcriptomic response of Huangjinya tea variety to different colored shading nets.

### 3.1 Different colored shading nets affect Huangjinya tea differently

Shading provides a form of protection for tea plants from sunburn by modifying the microenvironment. The practical use of shading nets in agriculture, and especially in tea industry, has been tested for several tea varieties (Zhang et al., 2022). It alters the chemical composition and sensory characteristics of tea. For example, Shofi and Betari (2023) reported that shading intensity impacted the sensory characteristics as well as bioactive compound composition in an Indonesian green tea variety “Assaica”. Our results suggest that shading with black (95% and 75%), red, yellow, and blue significantly affected the H1B3L, L1B3L, DNSB, and RNSB. It validates that Huangjinya tea is sensitive to sunlight (Figure 1). Earlier work on shading treatments in tea has shown that shading treatment resulted in increased leaf blade length, branch length, and leaf water content of Matcha green tea (Sano et al., 2018; Chen et al., 2022). These changes have been associated with carbon and nitrogen metabolism (Shao et al., 2022). The results that both black shading nets had lowest H1B3L and L1B3L indicate that colored shading nets are better for tea plants. However, considerably higher DNSB and RNSB observed for 95% black treatment, could be attributed to changes in chlorophyll content and oxidative damage (Sano et al., 2020). Among the different colored shades, Red showed the least DNSB and RNSB (Figure 1). Previously, it has been shown that tea plants exhibit higher plant growth indexes in response to Red shading nets compared to black and blue (Zhang et al., 2022). This is possibly due to the fact that growing under Red shading has a different spectral composition, and modifies the radiation intensity and UV intensity. As a consequence, this promotes vegetative growth and pigment synthesis (Kong and Nemali, 2021), as indicated by elevated ribulose-5P accumulation in the Red treatment and heightened expression of genes enriched in oxidative phosphorylation, photosynthesis, antenna proteins, and carotenoid biosynthesis pathways. This is also visible from the color differences in the leaves grown under normal sunlight conditions and those grown under colored shading nets (Figure 1B). Based on these observations, it can be concluded that Red shading nets prove beneficial in safeguarding the Huangjinya tea variety from sunburn. Moreover, Red shading nets exhibit superior efficacy compared to blue, yellow, and black shading options. Growing Huangjinya tea under Red shading affects amino acid, flavonoid, and lipid contents.

Previous work on growing tea plants under Red shading nets indicated changes in metabolome content compared to the plants grown under normal sunlight conditions (Zhang et al., 2014; Zhang et al., 2022). The Red shade net reduced the amino acid and derivatives, flavonoids, and alkaloids, content in Huangjinya tea leaves. Earlier research has shown higher amino acid contents in response to Red shade nets (Zhang et al., 2022). The up-accumulation of val-trp, L-phenylalanyl-Lphenylalanine, and L-homomethionine is consistent with the previous work (Matsunaga et al., 2016). However, the downregulation of several other amino acids is also a new observation. Insights drawn from the integrated transcriptome data suggest a comparatively higher number of genes involved in various amino acid metabolism pathways, as reflected in Supplementary Table S5. This observation hints at the potential mechanisms behind these findings. A thorough exploration of amino acid metabolism can provide a detailed understanding of the underlying processes. Other than amino acids, the most noticeable change was the flavonoid content changes in response to the Red treatment (Figures 3D, 8). Earlier research has also signified that the flavonoids are sensitive to shading treatment. For example, Ye et al. (2021) reported that light intensity and spectral composition in the field alter the composition of flavonoids in tea. Our results suggest that several transcripts of the key genes including *CAD, 4CL, HCT, FS*, *F3′H*, and *UGT* were downregulated in response to growing Huangjinya tea under Red shading nets (Figure 8). The flavonoid biosynthesis pathway is present downstream of the phenylpropanoid biosynthesis pathway. A change in the expression of phenylpropanoid pathway will directly affect it (Ye et al., 2021). The upregulation of several other transcripts involved in these two pathways, i.e., *PAL, HCT, CH’H, ANS, COMT, UGT,* and *ANS* is understandable from the perspective of catechins, anthocyanins, and lignans and coumarins as we noted upregulation of several metabolites related to these compounds (Supplementary Table S2). Wheat research has highlighted similar findings where the use of red light increased lignin content (Li et al., 2021). Other than flavonoids, lipids (free fatty acids) are also found in green tea leaves, though their relative content is lower (Cui et al., 2023). They have critical roles in cellular membrane structure and physiological activities. The results that free fatty acid content was significant in Red treatment, is consistent with the expressions of several transcripts annotated as *TL-SDP* and *LOX21* (Supplementary Table S2; Figure 6). *TL-SDP* gene can be induced by light, and the fatty acids derived from its activity are required for stomata opening (McLachlan et al., 2016). However, based on transcript expression changes in response to Red treatment, it is premature to deduce that a similar mechanism is functional in Huangjinya leaves. The LOX’s catalyze the hydroperoxidation of polyunsaturated fatty acids. It can also act as natural flavoring agent as oxidation by LOX produces aroma (Singh, et al., 2022). Nevertheless, the upregulation of several genes involved in lipid metabolism related pathways indicates Red shading nets can induce lipid changes in Golden bud tea.

### 3.2 Growing Huangjinya tea under Red shading affects Jasmonic acid biosynthesis

Shading nets of different colors can induce changes in physiological processes that are related to light. The physiological processes in plants are associated with phytohormone concentration changes and signaling (Gálvez, Albacete, del Amor and López-Marín, 2020). The observation that JA and methyl jasmonate increased in response to growing Huangjinya tea under Red shade aligns with the expression changes in *LOX21, AOS, AOC, OPR, OPCL1, KAT1*, and *JMT2* (Supplementary Table S2; Figure 6). Since red light induces stomatal opening, in relation to the changes in fatty acid biosynthesis, which is linked with the increased JA content (Zhu et al., 2020). In some plant species, the accumulation of flavonoids is inhibited in response to methyl jasmonate treatment (Yamamoto et al., 2020). This is consistent with reduced flavonoids and increased JA in response to growing tea plants under Red shade nets (Supplementary Table S2). It is also possible that growing Huangjinya tea under Red shade improves JA biosynthesis, stomatal conductance, and consequently, the overall performance of the plants compared to CK conditions. This proposition is supported by the known role of JA and the fact that we observed upregulation of 27 DEGs related to GO annotations (Supplementary Table S4). Our results suggest an interplay of fatty acids and JA may be functional to improve the Huangjinya tea performance by growing under Red shade nets.

### 3.3 Growing Huangjinya tea under Red shading alters catechin and caffeine biosynthesis

The large-scale changes in flavonoid and amino acid accumulation (discussed above) are also relevant from the catechin and caffeine accumulation perspective. Our results suggest that dihydrodicatechin A, gallic acid are consistent with the observed changes in flavonoids (Figure 3). Catechin biosynthesis is part of flavonoid biosynthesis pathway (Xiong et al., 2013). Dehydrodicatechin A is an oxidative dimer as a result of oxidation of flavan-3-ols (He et al., 2008). The findings indicate that under Red shading net conditions, there is a reduction in flavanols and flavonols content, as shown in Figure 3D. Additionally, there is an upregulation of DFR transcripts, and an increase in dehydrodicatechin A, suggesting an elevated level of oxidation (Wang et al., 2012). DFR converts dihydromyricetin to leucocyanidin, which is ultimately converted to catechin (Petrussa et al., 2013). Furthermore, the alterations in expression mentioned above concerning phenylpropanoid and flavonoid biosynthesis are pertinent. These outcomes align with findings reported earlier (Zhang et al., 2022). However, to deploy Red shading net to control the catechins biosynthesis in Huangjinya tea, the intensity and duration of the treatment will need to be further explored before a recommendation can be made (Shofi and Betari, 2023).

The presence of caffeine in green tea offers advantages as well as disadvantages (Vuong and Roach, 2014). It is known that shading can increase caffeine content in tea leaves (Chen et al., 2022). Red shading net increased caffeine content in tea grown in different seasons compared to control (Zhang et al., 2022), which is also consistent with our results. The metabolome profile clearly showed the reduction in xanthosine, 7-methylxanthine, and 1,7-dimethylxanthine, indicating increased caffeine biosynthesis (Figure 3A). However, the absence of DEGs related to this alteration indicates that these modifications might be due to differential regulation of other upstream pathways. Xanthosine is biosynthesized in purine metabolism pathway (Pang et al., 2012). The increased expression of *apyrase*, a gene associated with guanosine 5′-diphosphate and guanosine 5′-monophosphate (Wu et al., 2007), and the concurrent decrease in 5′-nucleotidase are interconnected. Xanthosine 5′-phosphate is converted to xanthosine by the action of 5′-nucleotidase (Ashihara et al., 2018) (Supplementary Table S4).

These results collectively indicate that growing Huangjinya tea under Red shading nets can influence caffein and catechins biosynthesis. Our results provide several candidate genes and pathways related to catechins and caffeine biosynthesis in Huangjinya tea under Red shading net treatment.

## 4 Conclusion

**FIGURE 10:**
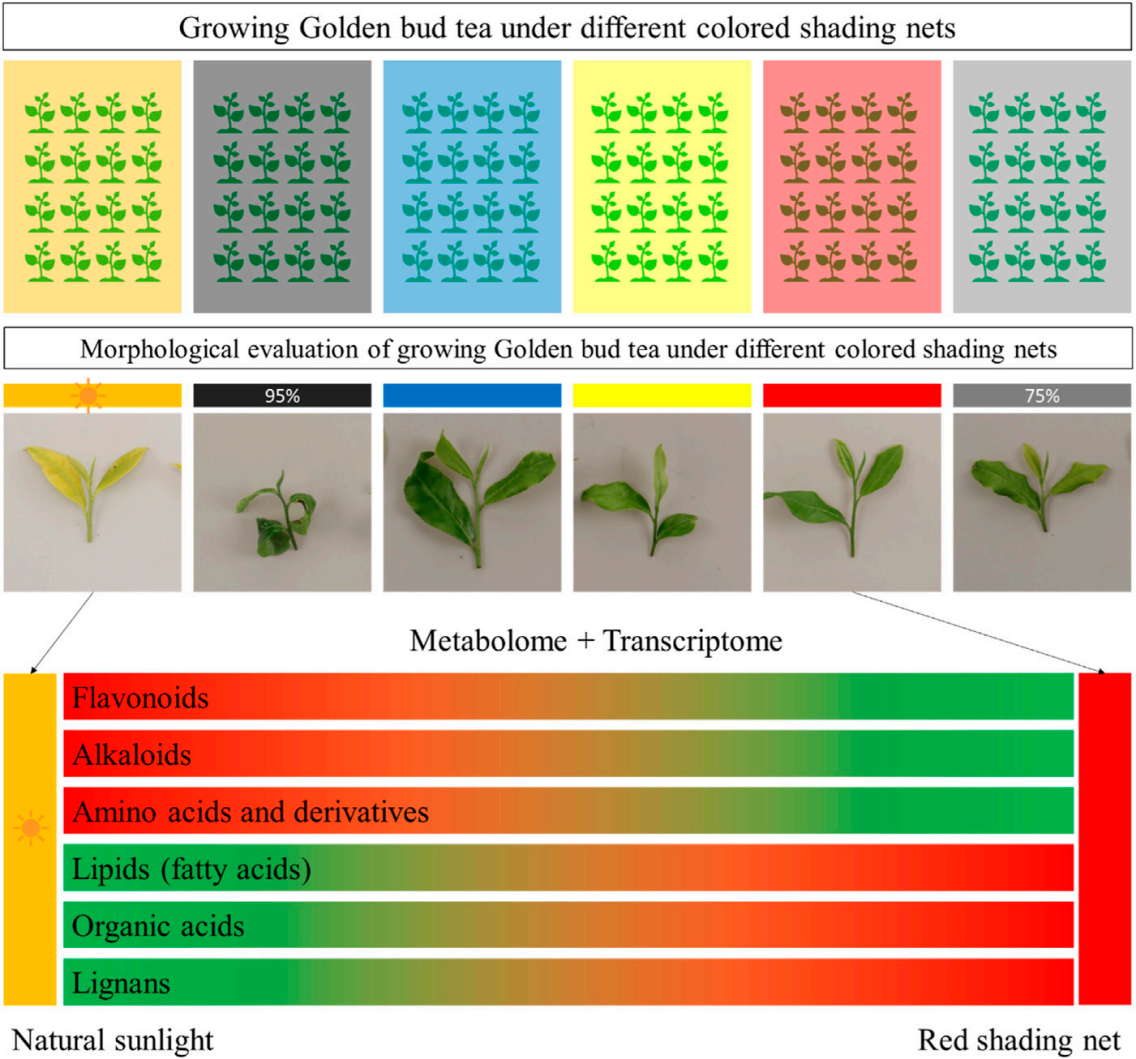
Effect of growing Huangjinya tea under different shading nets. Huangjinya tea one bud and three leaves showed minimum degree and rate of new shoot burn under Red shading net. The transcriptome and metabolome profile showed that flavonoids, alkaloids, and amino acids and derivatives content decreased, whereas, lipids, organic acids, and lignans content increased under red shading net. The green and red color indicate low and up accumulation/regulation of the metabolites.

In this study, we grew Huangjinya tea (a light sensitive tea variety used for green tea) under different colored shading nets for short period of time (20 days). The morphological parameters indicated that growing under Red shading nets proved to be less damaging for the tea plants. By comparing metabolome profile of the tea plants grown under Red shading nets with CK, we conclude that flavonoids, alkaloids, and amino acids and derivatives were negatively affected. However, lipids, organic acids, and lignans were positively affected, i.e., their content increased in response to growing tea plants under red shading nets. The transcriptome profiling highlighted that expression changes in phenylpropanoid, flavonoids, and related pathways are associated with the observed metabolome changes. Whereas, it is possible that increased jasmonic acid biosynthesis, together with fatty acids biosynthesis, is associated with the improved performance of Huangjinya tea under red shading nets (Figure 10). These results imply that in future the Red shading nets are useful to grow Huangjinya plants. Further experiments on the long-term usage of the Red shading net is needed.

## 5 Materials and methods

Huangjinya tea plants were grown for 6 years in the natural environment at the tea plant germplasm resource nursery of Zhejiang, Lishui Institute of Agricultural and Forestry Sciences, Lishui City, Zhejiang Province, China (28°35 N, 119°23 E, 133–142 m above sea level).

Different shading nets were used to treat tea plants (Figure 1A). Based on the color and type of shading net, the experiment was divided into six groups: red, yellow, blue, black (75% shading rate), black (95% shading rate), and CK (without shading treatment). The experiment was performed in three replicates where each replication block was sized at 10 m × 5 m. Shading treatment began on 31^st^ May 2023. After 20 days of shading, the degree of leaf burns and growth-related physiological indicators were investigated. For an estimation of the rate of new shoots burn (RNSB) in response to light or shading, 20 samples of one bud + three leaves (1B3L) from the branch apex were selected from each shading treatment and were divided into 5 sets of replicates. For the evaluation of the degree of new shoot burn (DNSB), we selected 10 samples of 1B3L from each experimental treatment. The following criteria were adopted for categorizing DNSB: (i) Leaf burns *p*<5% is Level 1; (ii) Leaf burns 5%<p<15% is Level 2; (iii) Leaf burns 15%<p<25% is Level 3; (iv) Leaf burns 25%<p<50% is Level 4; (v) Leaf burns 50%<p is Level 5. For estimating the weight of 100 1B3L (H1B3L), 100 samples of 1B3L were selected from each experimental treatment, and the data were recorded in triplicates. To measure the length of 1B3L (L1B3L), a total of 30 samples of 1B3L were selected from each experimental treatment (in triplicates), and measured the length from the base of the bud to the top of leaves (accurate up to 0.1cm, 3 replicates).

### 5.1 Metabolome sample preparation, extraction, and detection

We employed freeze-drying with a Scientz-100F lyophilizer, followed by grinding at 30 Hz for 1.5 min using an MM 400 Retsch grinder. We weighed 50 mg of sample powder and mixed it with 70% methanolic aqueous (pre-cooled to −20°C) internal standard extract (1,200 μL). After vortexing the mixture for 30 s (6 times, once every 30 min), we centrifuged it at 12,000 rpm for 3 min. Finally, we removed the supernatant followed by filtration of sample through a microporous membrane (0.22 μm). Finally, the filtrate was ready for further analysis through UPLC-MS/MS.

A UPLC-ESI-MS/MS system was employed to analyze the sample extracts. The system was equipped with an ExionLC™ AD and tandem-mass-spectrometry (available at https://sciex.com.cn/). Standard analytical conditions were used for widely targeted metabolomics analysis (Chen et al., 2013). The UPLC involved an Agilent SB-C18 column (diameter 2.1 mm, length 100 mm, particles 1.8 µm), a mobile phase as solvent A; pure water containing 0.1% formic acid), and solvent B (acetonitrile containing 0.1% formic acid). The analysis employed following program (gradient): starting with A (95%) and B (5%), transitioning to B (95%) and A (5%) over 9 min, maintaining this condition for 01 min. Afterward, the composition was altered to A (95%) and B (5%) in 1.1 min. This condition was sustained for another 2.9 min. The rate of flow was set at 0.35 mL/min and the temperature of column oven was set at 40°C. The volume of injection was set at 2 μL. The direction of effluent was connected to a QTRAP-MS (ESI-triple quadrupole-linear ion trap system). Following parameters were included in ESI source operation: 550°C source temperature, 5500 V of ion spray (IS, in positive ion mode)/-4500 V (negative ion mode), 50 psi (ion source gas I or GSI), 60 psi (ion source gas II or GSII), and 25 psi (curtain gas or CUR). CAD (Collision-activated dissociation) was set to high, and the collision gas (nitrogen) was set to medium levels to acquire QQQ scans as MRM experiments. CE (collision energy) and DP (declustering potential) for MRM transitions were optimized individually. During each period, based on eluted metabolites, specific MRM transitions were supervised.

Unsupervised Principal Component Analysis (PCA) was conducted using the “prcomp” function in the R programming environment (www.r-project.org). Pearson correlation coefficients (PCC) among samples were computed using the “cor” function in R, and the results were visualized as heatmaps. Hierarchical clustering analysis was performed in R using “ComlexHeatmap” package. Differential metabolites were identified based on VIP (Variable Importance in Projection) scores (VIP > 1) and the absolute Log2 Fold Change (|Log2 FC| ≥ 1.0). Metabolite annotation and mapping were performed using the KEGG Compound database (http://www.kegg.jp/kegg/compound/), and Pathway database (http://www.kegg.jp/kegg/pathway.html), respectively. When comparing the contents of individual of combined metabolites in a class/pathway, the Student’s t-test was applied (De Winter, 2019).

### 5.2 Transcriptome analysis

#### 5.2.1 Sample collection, RNA quantification, and qualification

RNA integrity and purity were verified using the RNA Nano 6000 Assay Kit of the Bioanalyzer 2100 system (Agilent Technologies, CA, USA) and NanoPhotometer^®^ spectrophotometer (IMPLEN, CA, USA), respectively. The Qubit^®^ RNA Assay Kit in Qubit^®^2.0 Flurometer (Life Technologies, CA, USA) was used to measure the concentration of RNA. A total amount of 1 µg RNA per sample was used to generate sequencing libraries using NEBNext^®^ UltraTM RNALibrary Prep Kit for Illumina^®^ (NEB, USA), and the Agilent Bioanalyzer 2100 system was used to assess the quality of library. The cBot Cluster Generation System using TruSeq PE Cluster Kit v3-cBot-HS (Illumia) was used for clustering analysis. The library preparations were sequenced on an Illumina platform (150 bp paired-end reads).

#### 5.2.2 Data analysis

The “fastp” program was used for filtering original sequencing data. Transcriptome assembly was performed using Trinity (https://github.com/trinityrnaseq/trinityrnaseq). Corset was used to regroup relevant transcripts into ‘gene’ clusters. The TransDecoder (https://github.com/TransDecoder/TransDecoder/wiki) was employed to identify candidate coding regions within transcript sequences generated by *de novo* RNA-Seq transcript assembly. Functional annotation of genes was performed using HMMER or diamond in following databases: Nr (NCBI non-redundant protein sequences) Swiss-Prot (A manually annotated and reviewed protein sequence database); Trembl(a computer annotated supplement to SWISS-PROT); GO (Gene Ontology); KOG/COG (COG: Clusters of Orthologous Groups of proteins; Pfam (Protein family); KOG: euKaryotic Ortholog Groups) and KEGG (Kyoto Encyclopedia of Genes and Genomes). Gene expression levels were estimated using RSEM package and Fragments Per Kilobase of transcript per Million mapped reads (FPKM) values. The DESeq2 was used to analyze the differential expression between the two groups. The *p*-value was corrected using the Benjamini & Hochberg method. The screening conditions for differentially expressed genes (DEGs) were |log 2 Fold Change| ≥ 1, and FDR (False Discovery Rate) < 0.05. Enrichment analysis utilized the hypergeometric test, employing the hypergeometric distribution test at the pathway level for KEGG and at the GO term level for Gene Ontology (GO).

To validate the RNA-seq data, changes in the gene expression of selected DEGs were analyzed via real-time quantitative RT-qPCR). The list of primers is presented in Supplementary Table S1.

### 5.3 Statistical analysis

The significant differences between means were performed with xlstat v.2021.5 with the least significant difference test at *p-value < 0.05.*

## Data Availability

The original contributions presented in the study are publicly available. This data can be found here: https://www.ncbi.nlm.nih.gov/bioproject/?term=PRJNA1044560.

## Supplementary material

SUPPLEMENTARY FIGURE S1 Differential metabolite clustering heatmap of KEGG pathways: The abscissa is the sample name, the ordinate is the differential metabolite, the different colors are the colors filled with different values obtained after normalization of different relative contents (red represents high content, green represents low content), the annotation bar above the heat map Corresponding to the sample group (Group), the dendrogram on the left side of the heat map represents the hierarchical clustering results of differential metabolites. The annotation bar on the right side of the cluster tree corresponds to the first-level classification of substances (Class). Different colors represent different substance categories. mws4002 (Dopamine), pme1002 (L-Tyramine), pme2598 (3,4-Dihydroxybenzeneacetic acid*), mws0180 (2,5-Dihydroxybenzoic acid; Gentisic Acid*), pme1292 (Homogentisic acid*), pme0220 (Methyl jasmonate), pme1654 (Jasmonic acid), Zmpn003368 (13S-Hydroxy-9Z,11E,15Z-octadecatrienoic acid), Zmjn003398 (2-Dodecenedioic acid), Zmyn004449 (9-Hydroxy-12-oxo-10(E),15(Z)-octadecadienoic acid), ZBN0231 (Vitexin-2″-O-glucoside*), Hmln002321 (Quercetin-3-O-(6″-O-malonyl)glucoside), mws0059 (Quercetin-3-O-rutinoside (Rutin)*), Lmsn002815 (Kaempferol-3-O-rutinoside(Nicotiflorin)*), mws0091 (Quercetin-3-O-glucoside (Isoquercitrin)), pme0088 (Luteolin (5,7,3′,4′-Tetrahydroxyflavone)*), mws0913 (Kaempferol-3-O-galactoside (Trifolin)*), mws0045 (Quercetin-3-O-rhamnoside(Quercitrin)).

SUPPLEMENTARY FIGURE S2 KEGG Classification of the differentially accumulated metabolites in Huangjinya gree tea grown under red shading net compared to the natural sunlight conditions.

## References

[B1] Ashihara H. Stasolla C. Fujimura T. Crozier A. (2018). Purine salvage in plants. Phytochemistry 147, 89–124. 10.1016/j.phytochem.2017.12.008 29306799

[B2] Astill C. Birch M. R. Dacombe C. Humphrey P. G. Martin P. T. (2001). Factors affecting the caffeine and polyphenol contents of black and green tea infusions. J. Agric. Food Chem. 49 (11), 5340–5347. 10.1021/jf010759+ 11714326

[B3] Chen J. Wu S. Dong F. Li J. Zeng L. Tang J. (2021). Mechanism underlying the shading-induced chlorophyll accumulation in tea leaves. Front. plant Sci. 12, 779819. 10.3389/fpls.2021.779819 34925423 PMC8675639

[B4] Chen W. Gong L. Guo Z. Wang W. Zhang H. Liu X. (2013). A novel integrated method for large-scale detection, identification, and quantification of widely targeted metabolites: application in the study of rice metabolomics. Mol. Plant 6 (6), 1769–1780. 10.1093/mp/sst080 23702596

[B5] Chen X. Ye K. Xu Y. Zhao Y. Zhao D. (2022). Effect of shading on the morphological, physiological, and biochemical characteristics as well as the transcriptome of matcha green tea. Int. J. Mol. Sci. 23 (22), 14169. 10.3390/ijms232214169 36430647 PMC9696345

[B6] Cui H. N. Gu H. W. Li Z. Q. Sun W. Ding B. Li Z. (2023). Integration of lipidomics and metabolomics approaches for the discrimination of harvest time of green tea in spring season by using UPLC-Triple-TOF/MS coupled with chemometrics. Front. Sustain. Food Syst. 7, 1119314. 10.3389/fsufs.2023.1119314

[B7] Deng Y. Lu S. (2017). Biosynthesis and regulation of phenylpropanoids in plants. CRC. Crit. Rev. Plant Sci. 36, 257–290. 10.1080/07352689.2017.1402852

[B8] De Winter J. C. (2019). Using the Student's t-test with extremely small sample sizes. Pract. Assess. Res. Eval. 18 (1), 10. 10.7275/e4r6-dj05

[B9] Elango T. Jeyaraj A. Dayalan H. Arul S. Govindadamy R. Prathap K. (2023). Influence of shading intensity on chlorophyll, carotenoid and metabolites biosynthesis to improve the quality of green tea: a review. Energy Nexus 12, 100241. 10.1016/j.nexus.2023.100241

[B10] Fang Z. T. Jin J. Ye Y. He W. Z. Shu Z. F. Shao J. N. (2022). Effects of different shading treatments on the biomass and Transcriptome profiles of tea leaves (Camellia sinensis L.) and the regulatory effect on phytohormone biosynthesis. Front. plant Sci. 13, 909765. 10.3389/fpls.2022.909765 35812958 PMC9266624

[B11] Gálvez A. Albacete A. del Amor F. M. López-Marín J. (2020). The Use of red shade nets improves growth in salinized pepper (Capsicum annuum L.) plants by regulating their ion homeostasis and hormone balance. Agronomy 10 (11), 1766. 10.3390/agronomy10111766

[B12] He F. Pan Q. H. Shi Y. Duan C. Q. (2008). Biosynthesis and genetic regulation of proanthocyanidins in plants. Molecules 13 (10), 2674–2703. 10.3390/molecules13102674 18971863 PMC6245171

[B13] Jin J. Lv Y. Q. He W. Z. Li D. Ye Y. Shu Z. F. (2021). Screening the key region of sunlight regulating the flavonoid profiles of young shoots in tea plants (Camellia sinensis L.) based on a field experiment. Molecules 26 (23), 7158. 10.3390/molecules26237158 34885740 PMC8659094

[B14] Kong Y. Nemali K. (2021). Blue and far-red light affect area and number of individual leaves to influence vegetative growth and pigment synthesis in lettuce. Front. plant Sci. 12, 667407. 10.3389/fpls.2021.667407 34305967 PMC8297648

[B15] Li C. Luo Y. Jin M. Sun S. Wang Z. Li Y. (2021). Response of lignin metabolism to light quality in wheat population. Front. plant Sci. 12, 729647. 10.3389/fpls.2021.729647 34589105 PMC8473876

[B16] Li Y. Jeyaraj A. Yu H. Wang Y. Ma Q. Chen X. (2020). Metabolic regulation profiling of carbon and nitrogen in tea plants [Camellia sinensis (L.) O. Kuntze] in response to shading. J. Agric. Food Chem. 68 (4), 961–974. 10.1021/acs.jafc.9b05858 31910000

[B17] Lin Z. Wei J. Hu Y. Pi D. Jiang M. Lang T. (2023). Caffeine synthesis and its mechanism and application by microbial degradation, A review. Foods 12 (14), 2721. 10.3390/foods12142721 37509813 PMC10380055

[B18] Matsunaga A. Sano T. Hirono Y. Horie H. (2016). Effects of various directly covered shading levels on chemical components in tea new shoots of the first flush. 茶業研究報告 122, 1–7. 10.5979/cha.2016.122_1

[B19] McLachlan D. H. Lan J. Geilfus C. M. Dodd A. N. Larson T. Baker A. (2016). The breakdown of stored triacylglycerols is required during light-induced stomatal opening. Curr. Biol. 26 (5), 707–712. 10.1016/j.cub.2016.01.019 26898465 PMC4791430

[B20] Pan S. Y. Nie Q. Tai H. C. Song X. L. Tong Y. F. Zhang L. J. F. (2022). Tea and tea drinking: China’s outstanding contributions to the mankind. Chin. Med. 17 (1), 27–40. 10.1186/s13020-022-00571-1 35193642 PMC8861626

[B21] Pang B. McFaline J. L. Burgis N. E. Dong M. Taghizadeh K. Sullivan M. R. (2012). Defects in purine nucleotide metabolism lead to substantial incorporation of xanthine and hypoxanthine into DNA and RNA. Proc. Natl. Acad. Sci. 109 (7), 2319–2324. 10.1073/pnas.1118455109 22308425 PMC3289290

[B22] Petrussa E. Braidot E. Zancani M. Peresson C. Bertolini A. Patui S. (2013). Plant flavonoids—biosynthesis, transport and involvement in stress responses. Int. J. Mol. Sci. 14 (7), 14950–14973. 10.3390/ijms140714950 23867610 PMC3742282

[B23] Reygaert W. C. (2017). An update on the health benefits of green tea. Beverages 3 (1), 6. 10.3390/beverages3010006

[B24] Sano S. Takemoto T. Ogihara A. Suzuki K. Masumura T. Satoh S. (2020). Stress responses of shade-treated tea leaves to high light exposure after removal of shading. Plants 9 (3), 302. 10.3390/plants9030302 32121552 PMC7154902

[B25] Sano T. Horie H. Matsunaga A. Hirono Y. (2018). Effect of shading intensity on morphological and color traits and on chemical components of new tea (Camellia sinensis L.) shoots under direct covering cultivation. J. Sci. Food Agric. 98 (15), 5666–5676. 10.1002/jsfa.9112 29722013

[B26] Shao C. Jiao H. Chen J. Zhang C. Liu J. Chen J. (2022). Carbon and nitrogen metabolism are jointly regulated during shading in roots and leaves of Camellia Sinensis. Front. plant Sci. 13, 894840. 10.3389/fpls.2022.894840 35498711 PMC9051521

[B27] Shin Y. H. Yang R. Shi Y. L. Li X. M. Fu Q. Y. Lu J. L. (2018). Light-sensitive albino tea plants and their characterization. HortScience 53 (2), 144–147. 10.21273/hortsci12633-17

[B28] Shofi V. E. Betari B. K. Supriyadi A. (2023). Effect shading intensity on color, chemical composition, and sensory evaluation of green tea (Camelia sinensis var Assamica). J. Saudi Soc. Agric. Sci. 22, 407–412. 10.1016/j.jssas.2023.03.006

[B29] Singh P. Arif Y. Miszczuk E. Bajguz A. Hayat S. (2022). Specific roles of lipoxygenases in development and responses to stress in plants. Plants 11 (7), 979. 10.3390/plants11070979 35406959 PMC9002551

[B30] Too J. C. Kinyanjui T. Wanyoko J. K. Wachira F. N. (2015). Effect of Sunlight Exposure and Different Withering Durations on Theanine Levels in Tea (<i&amp;gt;Camellia sinensis&amp;lt;/i&amp;gt;). Food Nutr. Sci. 6 (11), 1014–1021. 10.4236/fns.2015.611105

[B31] Vuong Q. V. Roach P. D. (2014). Caffeine in green tea: its removal and isolation. Sep. Purif. Rev. 43 (2), 155–174. 10.1080/15422119.2013.771127

[B32] Wang F. Yan J. Chen X. Jiang C. Meng S. Liu Y. (2019). Light regulation of chlorophyll biosynthesis in plants. Acta Hortic. Sin. 46 (5), 975–994. 10.16420/j.issn.0513-353x.2018-0799

[B33] Wang Y. Gao L. Shan Y. Liu Y. Tian Y. Xia T. (2012). Influence of shade on flavonoid biosynthesis in tea (Camellia sinensis (L.) O. Kuntze). Sci. Hortic. 141, 7–16. 10.1016/j.scienta.2012.04.013

[B34] Wang Kairong L. M. Yuerong L. Zhang L. Shen L. Wang S. (2008). Research on the breeding of new tea variety golden bud. Chin. Tea (4), 21–23. [茶树新品种黄金芽选育研究].

[B35] Wu J. Steinebrunner I. Sun Y. Butterfield T. Torres J. Arnold D. (2007). Apyrases (nucleoside triphosphate-diphosphohydrolases) play a key role in growth control in Arabidopsis. Plant Physiol. 144 (2), 961–975. 10.1104/pp.107.097568 17434987 PMC1914212

[B36] Xiang P. Zhu Q. Tukhvatshin M. Cheng B. Tan M. Liu J. (2021). Light control of catechin accumulation is mediated by photosynthetic capacity in tea plant (Camellia sinensis). BMC Plant Biol. 21 (1), 478–513. 10.1186/s12870-021-03260-7 34670494 PMC8527772

[B37] Xiong L. Li J. Li Y. Yuan L. Liu S. Huang J. a. (2013). Dynamic changes in catechin levels and catechin biosynthesis-related gene expression in albino tea plants (Camellia sinensis L.). Plant Physiology Biochem. 71, 132–143. 10.1016/j.plaphy.2013.06.019 23911731

[B38] Yamamoto R. Ma G. Zhang L. Hirai M. Yahata M. Yamawaki K. (2020). Effects of salicylic acid and methyl jasmonate treatments on flavonoid and carotenoid accumulation in the juice sacs of satsuma Mandarin *in vitro* . Appl. Sci. 10 (24), 8916. 10.3390/app10248916

[B39] Ye J. H. Lv Y. Q. Liu S. R. Jin J. Wang Y. F. Wei C. L. (2021). Effects of light intensity and spectral composition on the transcriptome profiles of leaves in shade grown tea plants (Camellia sinensis L.) and regulatory network of flavonoid biosynthesis. Molecules 26 (19), 5836. 10.3390/molecules26195836 34641378 PMC8510202

[B40] Yu Z. Liao Y. Zeng L. Dong F. Watanabe N. Yang Z. (2020). Transformation of catechins into theaflavins by upregulation of CsPPO3 in preharvest tea (Camellia sinensis) leaves exposed to shading treatment. Food Res. Int. 129, 108842. 10.1016/j.foodres.2019.108842 32036878

[B41] Zeng L. Watanabe N. Yang Z. (2019). Understanding the biosyntheses and stress response mechanisms of aroma compounds in tea (Camellia sinensis) to safely and effectively improve tea aroma. Crit. Rev. Food Sci. Nutr. 59 (14), 2321–2334. 10.1080/10408398.2018.1506907 30277806

[B42] Zhang Q. Bi G. Li T. Wang Q. Xing Z. LeCompte J. (2022). Color shade nets affect plant growth and seasonal leaf quality of Camellia sinensis grown in Mississippi, the United States. Front. Nutr. 9, 786421. 10.3389/fnut.2022.786421 35187030 PMC8847693

[B43] Zhang Q. Shi Y. Ma L. Yi X. Ruan J. (2014). Metabolomic analysis using ultra-performance liquid chromatography-quadrupole-time of flight mass spectrometry (UPLC-Q-TOF MS) uncovers the effects of light intensity and temperature under shading treatments on the metabolites in tea. PloS one 9 (11), e112572. 10.1371/journal.pone.0112572 25390340 PMC4229221

[B44] Zhao S. Cheng H. Xu P. Wang Y. (2023). Regulation of biosynthesis of the main flavor-contributing metabolites in tea plant (Camellia sinensis): a review. Crit. Rev. Food Sci. Nutr. 63 (30), 10520–10535. 10.1080/10408398.2022.2078787 35608014

[B45] Zhu M. Geng S. Chakravorty D. Guan Q. Chen S. Assmann S. M. (2020). Metabolomics of red-light-induced stomatal opening in *Arabidopsis thaliana*: coupling with abscisic acid and jasmonic acid metabolism. Plant J. 101 (6), 1331–1348. 10.1111/tpj.14594 31677315

[B46] Zoratti L. Karppinen K. Luengo Escobar A. Häggman H. Jaakola L. (2014). Light-controlled flavonoid biosynthesis in fruits. Front. plant Sci. 5, 534. 10.3389/fpls.2014.00534 25346743 PMC4191440

