# Supplementary figures and images for: Combined metabolome and transcriptome analyses reveal that growing under Red shade affects secondary metabolite content in Huangjinya green tea
Source: Front Genet. 2024 Apr 10;15:1365243. doi: 10.3389/fgene.2024.1365243 (PMC11039865; doi:10.3389/fgene.2024.1365243)

## Slide 1
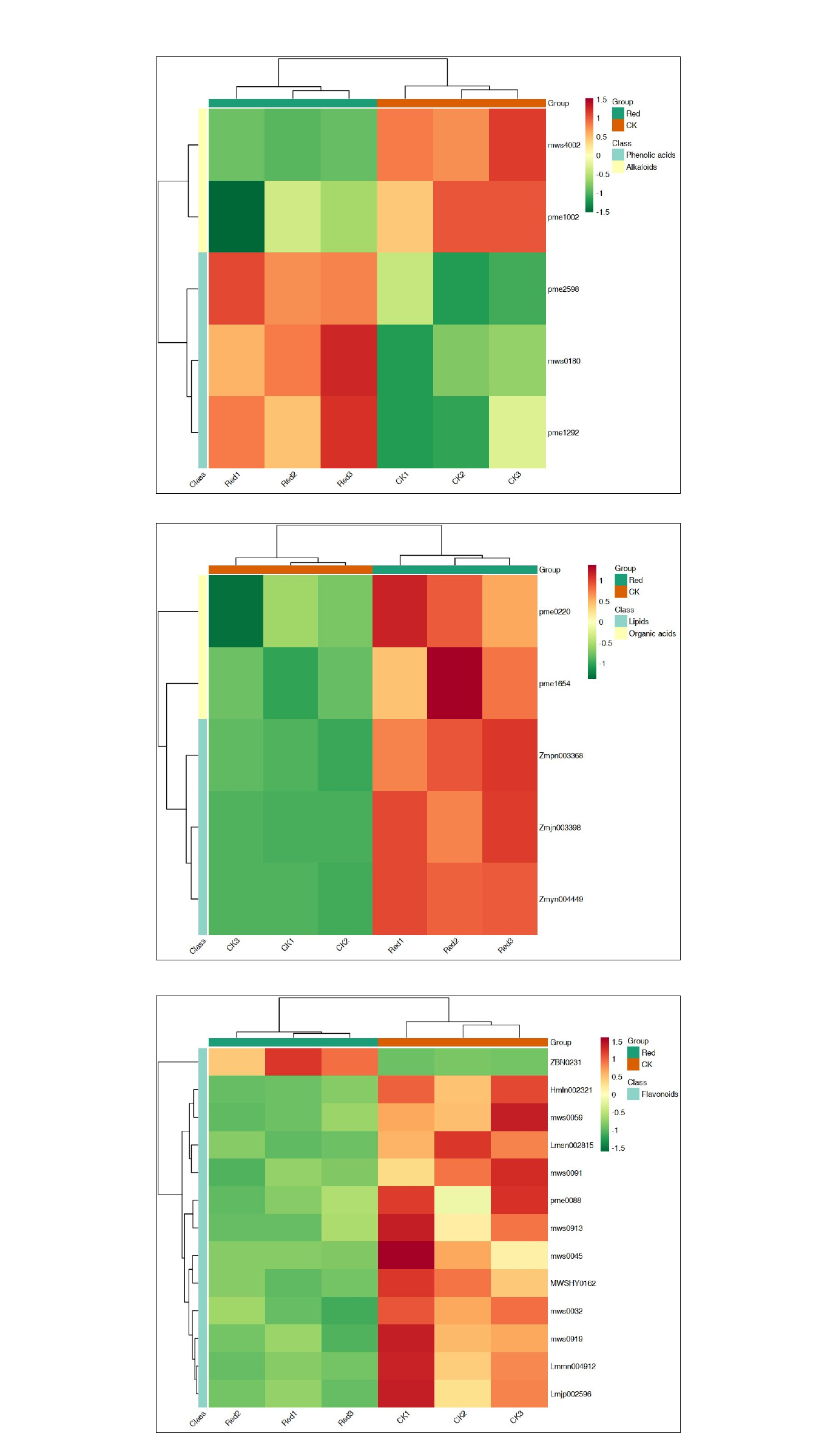

Supplement: Supplementary file 1 [file Presentation1.PPTX]
