# Supplementary material for: Combined metabolome and transcriptome analyses reveal that growing under Red shade affects secondary metabolite content in Huangjinya green tea
Source: Front Genet. 2024 Apr 10;15:1365243. doi: 10.3389/fgene.2024.1365243 (PMC11039865; doi:10.3389/fgene.2024.1365243)
Supplement: Supplementary file 2 [file DataSheet1.PDF]

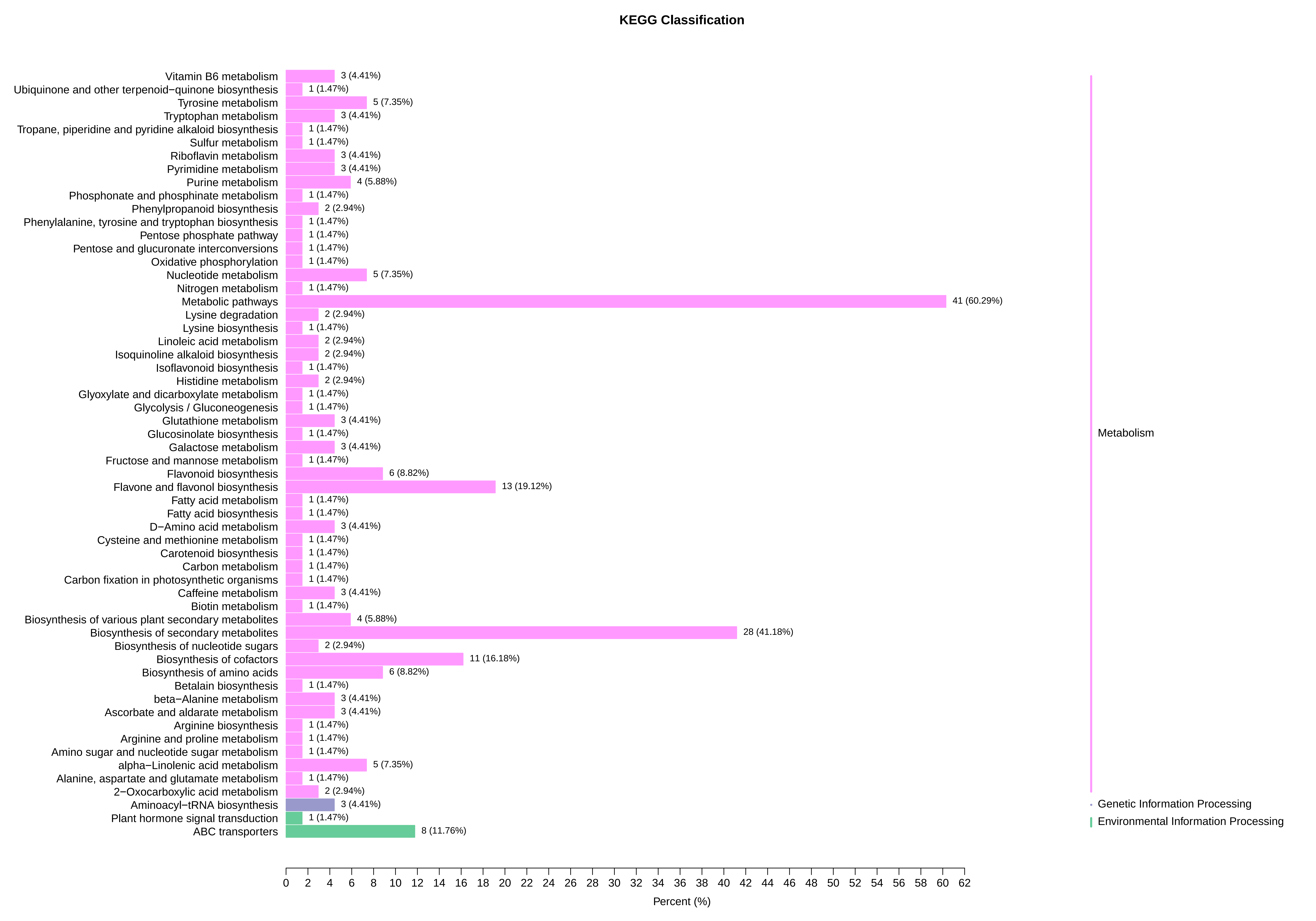
KEGG Classification
Vitamin B6 metabolism 3 (4.41%)
Ubiquinone and other terpenoid−quinone biosynthesis 1 (1.47%)
Tyrosine metabolism 5 (7.35%)
Tryptophan metabolism 3 (4.41%)
Tropane, piperidine and pyridine alkaloid biosynthesis 1 (1.47%)
Sulfur metabolism 1 (1.47%)
Riboflavin metabolism 3 (4.41%)
Pyrimidine metabolism 3 (4.41%)
Purine metabolism 4 (5.88%)
Phosphonate and phosphinate metabolism 1 (1.47%)
Phenylpropanoid biosynthesis 2 (2.94%)
Phenylalanine, tyrosine and tryptophan biosynthesis 1 (1.47%)
Pentose phosphate pathway 1 (1.47%)
Pentose and glucuronate interconversions 1 (1.47%)
Oxidative phosphorylation 1 (1.47%)
Nucleotide metabolism 5 (7.35%)
Nitrogen metabolism 1 (1.47%)
Metabolic pathways 41 (60.29%)
Lysine degradation 2 (2.94%)
Lysine biosynthesis 1 (1.47%)
Linoleic acid metabolism 2 (2.94%)
Isoquinoline alkaloid biosynthesis 2 (2.94%)
Isoflavonoid biosynthesis 1 (1.47%)
Histidine metabolism 2 (2.94%)
Glyoxylate and dicarboxylate metabolism 1 (1.47%)
Glycolysis / Gluconeogenesis 1 (1.47%)
Glutathione metabolism 3 (4.41%)
Glucosinolate biosynthesis 1 (1.47%)
Galactose metabolism 3 (4.41%)
Fructose and mannose metabolism 1 (1.47%)
Flavonoid biosynthesis 6 (8.82%)
Flavone and flavonol biosynthesis 13 (19.12%)
Fatty acid metabolism 1 (1.47%)
Fatty acid biosynthesis 1 (1.47%)
D−Amino acid metabolism 3 (4.41%)
Cysteine and methionine metabolism 1 (1.47%)
Carotenoid biosynthesis 1 (1.47%)
Carbon metabolism 1 (1.47%)
Carbon fixation in photosynthetic organisms 1 (1.47%)
Caffeine metabolism 3 (4.41%)
Biotin metabolism 1 (1.47%)
Biosynthesis of various plant secondary metabolites 4 (5.88%)
Biosynthesis of secondary metabolites 28 (41.18%)
Biosynthesis of nucleotide sugars 2 (2.94%)
Biosynthesis of cofactors 11 (16.18%)
Biosynthesis of amino acids 6 (8.82%)
Betalain biosynthesis 1 (1.47%)
beta−Alanine metabolism 3 (4.41%)
Ascorbate and aldarate metabolism 3 (4.41%)
Arginine biosynthesis 1 (1.47%)
Arginine and proline metabolism 1 (1.47%)
Amino sugar and nucleotide sugar metabolism 1 (1.47%)
alpha−Linolenic acid metabolism 5 (7.35%)
Alanine, aspartate and glutamate metabolism 1 (1.47%)
2−Oxocarboxylic acid metabolism 2 (2.94%)
Aminoacyl−tRNA biosynthesis 3 (4.41%)
Plant hormone signal transduction 1 (1.47%)
ABC transporters 8 (11.76%)
0 2 4 6 8 10 12 14 16 18 20 22 24 26 28 30 32 34 36 38 40 42 44 46 48 50 52 54 56 58 60 62
Percent (%)
Metabolism
Genetic Information Processing
Environmental Information Processing
